# Ferroptosis: challenges and opportunities for nanomaterials in cancer therapy

**DOI:** 10.1093/rb/rbad004

**Published:** 2023-01-20

**Authors:** Qiaolin Liu, Yuliang Zhao, Huige Zhou, Chunying Chen

**Affiliations:** Henan Institutes of Advanced Technology, Zhengzhou University, Zhengzhou 450052, China; CAS Key Laboratory for Biomedical Effects of Nanoparticles and Nanosafety & CAS Center for Excellence in Nanoscience, National Center for Nanoscience and Technology, Beijing 100190, China; CAS Key Laboratory for Biomedical Effects of Nanoparticles and Nanosafety & CAS Center for Excellence in Nanoscience, National Center for Nanoscience and Technology, Beijing 100190, China; University of Chinese Academy of Sciences, Beijing 100049, China; Research Unit of Nanoscience and Technology, Chinese Academy of Medical Sciences, Beijing 100039, China; The GBA National Institute for Nanotechnology Innovation, Guangzhou 510700, Guangdong, China; CAS Key Laboratory for Biomedical Effects of Nanoparticles and Nanosafety & CAS Center for Excellence in Nanoscience, National Center for Nanoscience and Technology, Beijing 100190, China; University of Chinese Academy of Sciences, Beijing 100049, China; Research Unit of Nanoscience and Technology, Chinese Academy of Medical Sciences, Beijing 100039, China; CAS Key Laboratory for Biomedical Effects of Nanoparticles and Nanosafety & CAS Center for Excellence in Nanoscience, National Center for Nanoscience and Technology, Beijing 100190, China; University of Chinese Academy of Sciences, Beijing 100049, China; Research Unit of Nanoscience and Technology, Chinese Academy of Medical Sciences, Beijing 100039, China; The GBA National Institute for Nanotechnology Innovation, Guangzhou 510700, Guangdong, China

**Keywords:** ferroptosis, reactive oxygen species, stimuli-responsive nanomaterials, cancer therapy

## Abstract

Ferroptosis, a completely new form of regulated cell death, is mainly caused by an imbalance between oxidative damage and reductive protection and has shown great anti-cancer potential. However, existing small-molecule ferroptosis inducers have various limitations, such as poor water solubility, drug resistance and low targeting ability, hindering their clinical applications. Nanotechnology provides new opportunities for ferroptosis-driven tumor therapy. Especially, stimuli-responsive nanomaterials stand out among others and have been widely researched because of their unique spatiotemporal control advantages. Therefore, it’s necessary to summarize the application of those stimuli-responsive nanomaterials in ferroptosis. Here, we describe the physiological feature of ferroptosis and illustrate the current challenges to induce ferroptosis for cancer therapy. Then, nanomaterials that induce ferroptosis are classified and elaborated according to the external and internal stimuli. Finally, the future perspectives in the field are proposed. We hope this review facilitates paving the way for the design of intelligent nano-ferroptosis inducers.

## Graphical Abstract

**Figure:**
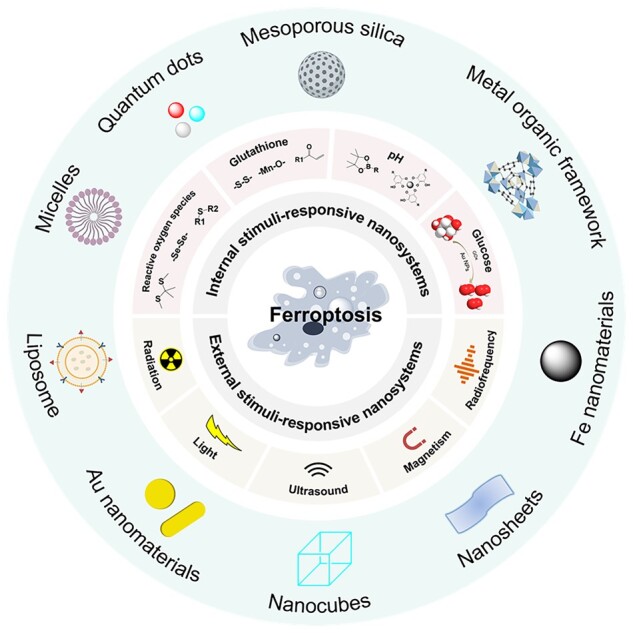

## Introduction

Cancer ranks among the deadliest diseases and threatens global health [1]. The heterogeneity, diversity and complexity of tumors pose a significant challenge to researchers. Therefore, therapeutic and diagnostic methods for cancer still need to be developed. Various novel cell death modes have been developed to explore their potential in the discovery of anti-cancer drugs, such as pyroptosis [2], apoptosis [3], autophagy [4] and ferroptosis [5]. Among the regulatable cell death modalities, ferroptosis, discovered in 2012 by Stockwell, is more popular and has received more attention than others because of its close relationship with tumors [6]. As a unique form of programmed cell death, ferroptosis relies on iron ions and reactive oxygen species (ROS) to induce lipid peroxidation, leading to cell death, completely distinguishing it from apoptosis, autophagy and necrosis at the morphological, molecular and genetic levels [7]. At the morphological level, the mitochondrial outer membrane of the ferroptotic cells ruptured and shrunk. Meanwhile, cristae inside mitochondria are reduced or even disappear, ultimately leading to their dysfunction [8]. At molecular level, iron ion accumulation, ROS over-production, glutathione (GSH) consumption and lipid peroxide level elevation are the main hallmarks of ferroptotic cells [9, 10]. At the genetic level, some genes have been identified as uniquely required for ferroptosis, including iron response element-binding protein 2, citrate synthase, acyl-CoA synthetase family member 2 and tetratricopeptide repeat domain 35 [11].

Since the term was first proposed, many efforts have been made to elucidate how to regulate ferroptosis in various scenarios to facilitate the application of this novel cell death modality in different diseases [12–16]. In recent years, growing evidence has proven that ferroptosis has received considerable attention from the cancer research community and shows great potential for cancer treatment [17–19]. Owing to the high metabolic features of tumors, most tumor cells exhibit a state of high oxidative stress and are relatively susceptible to ferroptosis, which are characteristic targets for tumor therapy [20]. Furthermore, ferroptosis potentiates the anti-tumor efficacy of multiple therapies, such as chemotherapy [21], radiotherapy (RT) [22, 23] and immunotherapy [24]. Ferroptosis evades the apoptotic pathway, which is promising for overcoming drug resistance in tumor therapy [25–28]. Therefore, it is promising to explore and develop methods that can enhance tumor therapeutic effects via regulation of ferroptosis.

Recently, research on ferroptosis inducers has increased exponentially, and various treatment methods for cancer via inducing ferroptosis have been widely reported, such as the use of small molecules (e.g. erastin, sorafenib [SRF], sulfasalazine [SAS]) and related genes (e.g. shMTHFD2 and shGPX4 plasmids) [11, 29]. However, the poor water solubility and instability of existing ferroptosis inducers greatly limit their further application in cancer therapy [30]. Fortunately, the designable, multi-functional and modifiable properties of nanomaterials provide diverse advantages to nanosystems of ferroptosis inducers, such as targeted delivery, stimuli-responsiveness and combination therapy [31–33]. In particular, owing to their precise spatiotemporal control capability, responsive nanosystems based on various external and internal stimuli are more advantageous for use in cancer therapy [34–36]. A series of responsive nanoplatforms have been designed and applied to treat cancer by triggering ferroptotic cell death [37, 38].

For the rapid development of ferroptosis and nanotechnology [39], it is necessary to summarize the various responsive nanoplatforms based on ferroptosis. Prior to this, the physiological features of ferroptosis and current challenges in inducing ferroptosis for cancer therapy are described in detail to gain a deeper understanding of ferroptosis. Then, ferroptosis induced by responsive nanomaterials is introduced, particularly focusing on the external stimuli-responsive nanosystems and internal stimuli-responsive nanosystems in this review. Finally, the observed challenges and future research directions of these responsive nanomaterials for inducing ferroptosis are discussed. It is hoped that this review will provide guidance for the design of nano-ferroptosis inducers from different perspectives.

## Physiological feature of ferroptosis

Along with the occurrence of ferroptosis, a series of physiological features reflects the molecular mechanism of ferroptosis and suggests its regulation [7]. Therefore, it is necessary to summarize the physiological characteristics of ferroptosis. In this section, we briefly summarize the physiological features of ferroptosis from the perspectives of abnormal iron metabolism, redox imbalance and excessive lipid peroxidation.

### Abnormal iron metabolism

Ferroptosis, as its name suggests, is an iron-mediated form of regulated cell death [40]. Iron plays an indispensable role in the induction of ferroptosis, and its intracellular concentration is an important indicator of ferroptosis [41]. Abnormal iron metabolism is a key signal and a major feature of ferroptosis [42]. Intracellular iron abundance is regulated by multiple pathways, and ferroptosis is promoted when the levels of the intracellular labile iron pool increase [43, 44]. Excess iron promotes ferroptosis by converting hydrogen peroxide (H_2_O_2_) to more toxic hydroxyl radicals (•OH) via the Fenton reaction [45, 46]. Furthermore, as an essential cofactor, iron governs ferroptosis by participating in the synthesis of lipid peroxidases, such as arachidonate lipoxygenase (ALOX) and cytochrome P450 oxidoreductase [47, 48].

### Redox imbalance

The key mechanism of ferroptosis involves antagonism between oxidative damage and antioxidant defense [49]. Ferroptosis is induced when there is an imbalance between ROS production and degradation during lipid peroxidation [50]. When ferroptosis occurs, significant production of intracellular ROS and an obvious depletion of antioxidant agents are detected, which is a prerequisite and the main feature of ferroptosis. There are many types of ROS, including hydrogen peroxide, hydroxyl radicals, superoxide anions ($O_{2}^{-}$), singlet oxygen (^1^O_2_), superoxide radical anions ($O_{2}^{•-}$) and hypochlorite anions (OCl^−^) [51, 52]. The glutathione peroxidase 4 (GPX4)–GSH system, the main intracellular antioxidant system, uses reduced GSH to detoxify lipid hydroperoxides via GPX4 (a phospholipid hydroperoxidase), which is the primary hindrance to ferroptosis [10]. In summary, ferroptosis is a mode of cell death caused by peroxidative toxicity induced by an intracellular redox imbalance [53].

### Excessive lipid peroxidation

The lethal accumulation of lipid peroxides is another dominant feature of ferroptosis, resulting from redox dysregulation and the direct executor of ferroptosis, initiating plasma membrane oxidative damage and eventual cell death [54]. Specifically, long-chain fatty acid-CoA ligase 4 (ACSL4) and lysophospholipid acyltransferase 5 promote the incorporation of polyunsaturated fatty acids (PUFAs) into phospholipids to form PUFA-containing phospholipids, which are vulnerable to ALOX and various ROS oxidation. This oxidation ultimately leads to disruption of the lipid bilayer and affects membrane function, eventually leading to ferroptosis [55].

## Current challenges to induce ferroptosis for cancer therapy

To date, research on the mechanism of ferroptosis and its application in cancer therapy has been progressively conducted. Various ferroptosis-inducing agents have been designed and prepared to achieve anti-tumor effects [49]. However, ferroptosis inducers still face huge challenges in clinical application, such as insufficient therapeutic effects, non-negligible drug side effects and unclear detection methods.

Due to poor water solubility, short half-life in the blood, and emergence of drug resistance, the therapeutic effectiveness of chemical inducers with small molecules is far from satisfactory [56]. For instance, erastin, a typical system xc^−^ inhibitor, has poor water solubility and unstable metabolism in the body, leading to uncontrollable side effects [57]. Deferoxamine, a Food and Drug Administration (FDA)-approved iron-chelating agent, is used to chelate iron ions and alleviate excessive ferroptosis damage in normal cells and tissues. However, its short half-life (∼15 min) severely limits its accumulation in cancer [58]. Drug resistance is another important factor that has the greatest impact on treatment efficacy and has appeared in SRF-mediated hepatocellular carcinoma therapy, attenuating its ferroptosis-inducing effect [59].

In addition, the side effects of available ferroptosis inducers are not negligible, which is a great challenge for the application of ferroptosis in cancer treatment. For example, cisplatin induces ferroptosis by downregulating intracellular GSH levels and suppressing GPX4 activity [21, 60]. However, its toxic effects on the kidneys and eyes restrict its widespread clinical application [61, 62]. Additionally, the low targeting ability of small-molecule inducers is one of the main causes of side effects. Some ferroptosis inducers, such as SRF and SAS, trigger ferroptosis by inhibiting the system xc—triggered downregulation of GSH and GPX4. Nevertheless, when GPX4 is downregulated in normal cells and tissues, some adverse effects occur, such as the induction of intestinal tumors and the death of antigen-specific CD4^+^ and CD8^+^ T cells [63, 64].

Moreover, the detection and analysis of ferroptosis-based therapeutic processes are important issues that need to be addressed. However, available imaging methods and novel diagnostic probes for monitoring this process have seldom been reported, which is a promising research direction for revealing the underlying mechanism of ferroptosis. From the current point of view, there is still a long way to go before ferroptosis can be used for cancer therapy in the clinic.

## Tailoring nanomaterials to regulate ferroptosis in cancer therapy

**Figure 1.**
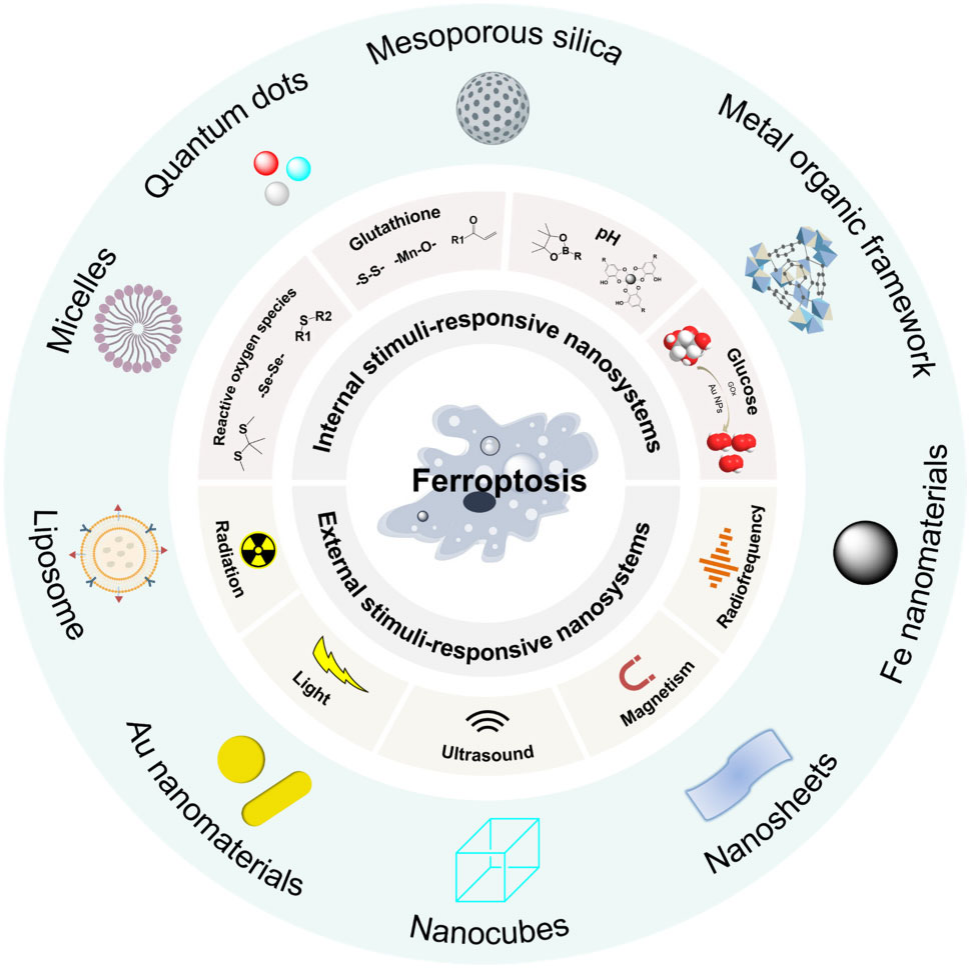
The category of stimuli-responsive nanomaterials to trigger ferroptosis.

Given the limited clinical applications of small-molecule ferroptosis inducers, tailoring nanomaterials to modulate ferroptosis in cancer therapy has attracted the attention of researchers. In the past few years, numerous studies have designed various functional nanomaterials to regulate ferroptosis [30]. Stimuli-responsive nanomaterials have been widely used for inducing ferroptosis due to their spatially and temporally controllable properties [65–67]. They are basically involved in two main categories: (i) external stimuli, such as radiation, light, ultrasound and magnetic field (MF); (ii) internal stimuli, such as ROS, GSH, pH and glucose, as illustrated in Fig. 1. In this section, a series of responsive nanomaterials that trigger ferroptosis are summarized, with an emphasis on external or internal stimuli-responsive nanosystems triggering ferroptosis for cancer therapy.

### External stimuli-responsive nanosystems for inducing ferroptosis

External stimuli-responsive nanomaterials are currently attracting the attention of researchers owing to their various beneficial features, such as their noninvasive nature and controllability [68]. More importantly, nanomaterials are controllably activated to exert anti-tumor effects upon external stimuli [69]. Many therapies are based on responses to external stimulation to achieve therapeutic effects. According to the external stimuli response sources, they are roughly divided into ionizing radiation-responsive RT, light-responsive photodynamic therapy (PDT) and ultrasound-responsive sonodynamic therapy (SDT). Herein, we summarize some ferroptosis-inducing nanomaterials based on different external stimulus-based therapies. Representative external stimuli-responsive nanosystems used to induce ferroptosis are listed in Table 1.

**Table 1.** The representative external stimuli-responsive nanosystems used for inducing ferroptosis

External stimulus	Nanosystem	Responsive composition	Core mechanism	*In vitro*/*in vivo* model	Ref
Radiation	AuFCSP MOFs	Au NPs	ROS accumulation, GSH depletion, GPX4 downregulation and lipid peroxidation	4T1 cells/BALB/c mice	[70]
	Bi_2_S_3_@mBi_x_Mn_y_O_z_	Bi_2_S_3_ NPs	ROS accumulation, GSH depletion, GPX4 downregulation and lipid peroxidation	4T1 cells/BALB/c mice	[71]
Light	SRF@Hb-Ce6	Ce6	ROS accumulation, GSH depletion, GPX4 downregulation and lipid peroxidation	4T1 cells/BALB/c mice	[72]
	Ir-g-C_3_N_4_	Ir(tpy)Cl_3_	ROS accumulation, GSH depletion, GPX4 downregulation and lipid peroxidation	MDA-MB-231 cells/Hela cells/Nu/Nu female mice	[73]
	Ir NPs@Biotin	Iridium (III) complexes	ROS accumulation, GSH depletion, GPX4 downregulation and lipid peroxidation	A549 cells/Nu/Nu female mice	[74]
	NSs@DCPy	DCPy	ROS accumulation, GSH depletion, GPX4 downregulation and lipid peroxidation	MC38 cells/BALB/c mice	[75]
	MP@CH/BSA	Ce6	ROS accumulation, GSH depletion, GPX4 downregulation and lipid peroxidation	4T1 cells/BALB/c mice	[76]
	TCLMs	Ce6	ROS accumulation, GSH depletion, GPX4 downregulation and lipid peroxidation	HepG2 and Huh7 cells/BALB/c mice	[77]
Ultrasound	Lipo-PpIX@Feru-moxytol	PpIX	ROS accumulation, GSH depletion, ACSL4 upregulation, GPX4 downregulation and lipid peroxidation	4T1 cells/BALB/c mice	[78]
	PpIX@MFc	PpIX	ROS accumulation, GSH depletion, thioredoxin depletion, GPX4 downregulation and lipid peroxidation	4T1 cells/BALB/c mice	[79]
	SAFE	Vp	ROS accumulation, GSH depletion, GPX4 downregulation and lipid peroxidation	4T1 cells/BALB/c mice	[80]
	FHPLP	PpIX	ROS accumulation and GSH depletion	Saos-2 cells/ICR mice and BALB/c mice	[81]
	Mn-MOF	Porphyrin	ROS accumulation, GSH depletion and GPX4 downregulation	4T1 cells and H22 cells/BALB/c mice	[82]
Magnetic field	HCSVs	Iron oxide nanocubes	ROS accumulation, GPX4 downregulation and lipid peroxidation	TRAMP-C1 cells/C57BL/6 mice	[83]

NPs, nanoparticles; MOF, metal organic framework; Ce6, Chlorin e6; PpIX, protoporphyrin IX; Vp, verteporfin.

#### Ionizing radiation stimuli-responsive nanosystems based on RT for inducing ferroptosis

RT, which exerts a therapeutic effect through ionizing radiation, is widely and frequently used clinically to treat various tumors [84–86]. Ionizing radiation generates ROS through the stimulation of oxidases and radiolysis of cellular water during RT, leading to cellular damage. Lei *et al*. [87] demonstrated that ionizing radiation-induced ferroptosis and tumor suppression by generating ROS and inducing the expression of ACSL4, which has a similar mechanism as the small-molecule inducers to induce ferroptosis. Other studies have shown that ferroptosis increased the sensitivity of tumor cells to RT [88–90]. Considering the close relationship between RT and ferroptosis, some nanomaterials have been designed for cancer treatment.

**Figure 2.**
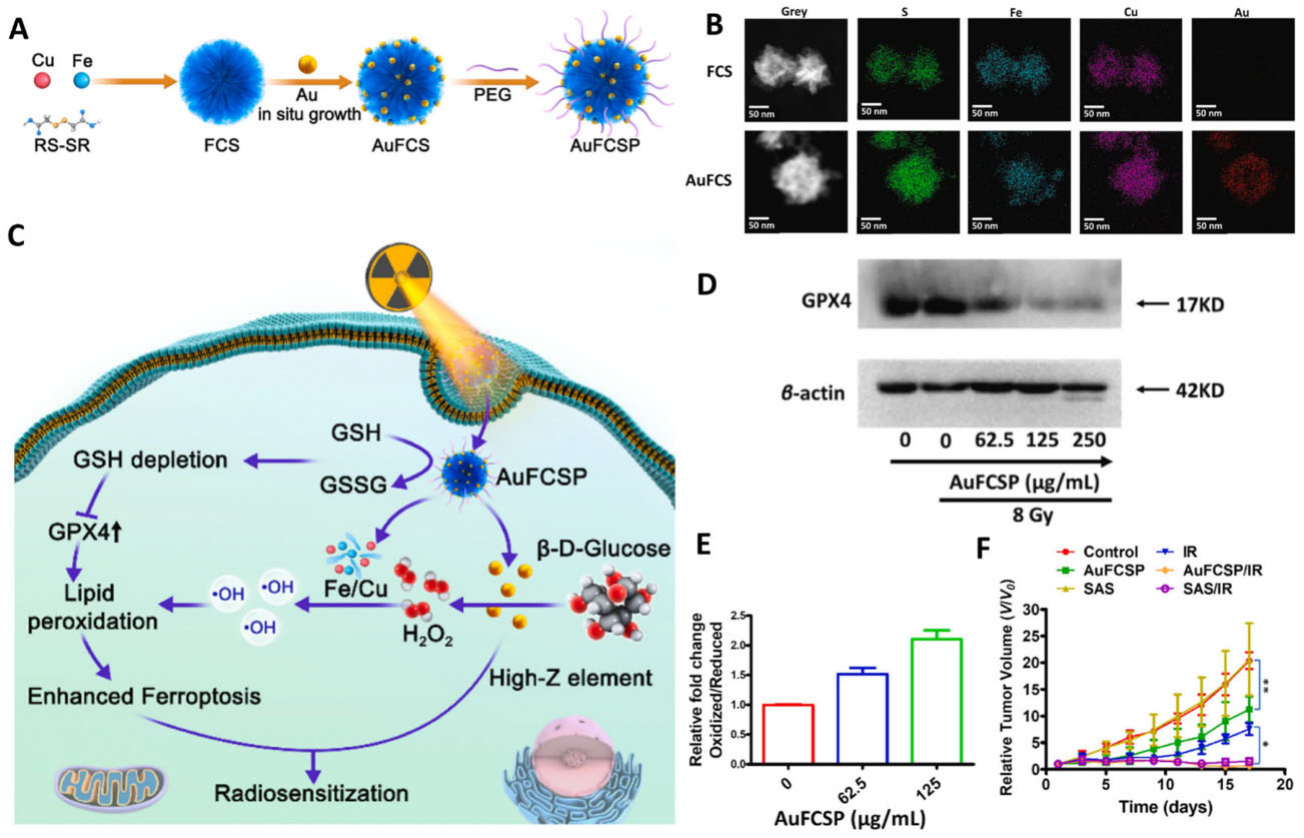
(**A**) Schematic illustration of the synthesis of TME self-activated AuFCSP MOFs. (**B**) TEM images and scanning transmission electron microscopy-energy dispersive spectrometer elemental maps of FCS MOFs and AuFCSP MOFs. (**C**) Schematic illustration of the TME self-activated Cascade catalytic AuFCSP MOFs for efficient ferroptosis and high-Z element-based breast cancer radiosesitization. (**D**) Western blotting analysis of GPX4 expression in 4T1 cell lines treated with different concentration AuFCSP MOFs. (**E**) Relative fold changes of lipid peroxidation in oxidized/reduced in 4T1 cells treated with different AuFCSP MOFs formulations. (**F**) Tumor weight from sacrificed animals at the end of the experiment, *P < 0.05, **P < 0.01, and ***P < 0.001. Adapted with permission from Ref. [70], © Elsevier 2022.

For the high-Z element properties of Au, gold-based nanomaterials have been widely used to respond ionizing radiation and enhance the therapeutic effect of RT. For example, Liang *et al*. [70] loaded Au NPs into the metal organic frameworks (MOFs) containing Fe and Cu dual ions bridged by dithiodiglycolic acid (Fig. 2A). The scanning transmission electron microscopy-energy dispersive spectrometer elemental maps of FCS MOFs and AuFCSP MOFs demonstrated the successfully loaded of Au NPs, Fe and Cu elements (Fig. 2B). For the existence of disulfide bond, Au NPs, Fe/Cu ions could be rapidly released in the tumor cells, accompanied by the depletion of GSH and downregulation of GPX4 (Fig. 2C and D). The released Au NPs catalyzed β-d-glucose to generate gluconic acid and H_2_O_2_ via their nanozyme properties, triggering Fenton and Fenton-like reactions with the released Fe/Cu ions to produce •OH and strengthen lipid peroxidation (Fig. 2C and E). The combination of those ferroptosis effects of AuFCSP MOFs and the ionizing radiation responsive ability of Au NPs achieves a significant inhibition of tumor growth (Fig. 2F).

Besides of Au, Bi is another safety and high-Z element responding to ionizing radiation. With the similar mechanism of AuFCSP MOFs, Hou *et al*. [71] fabricated a ‘yolk-shell’-like nanosystem (Bi_2_S_3_@mBi_x_Mn_y_O_z_), integrating the radiant energy deposition ability of Bi_2_S_3_ nanorods, the ferroptosis triggering ability of the Mn ions and the chemotherapeutic effect of doxorubicin (DOX). Results of *in vitro* and *in vivo* manifested that this combination strategy holds great promise to acquire the synergistic enhanced therapeutic effect.

#### Light stimuli-responsive nanosystems based on PDT for inducing ferroptosis

**Figure 3.**
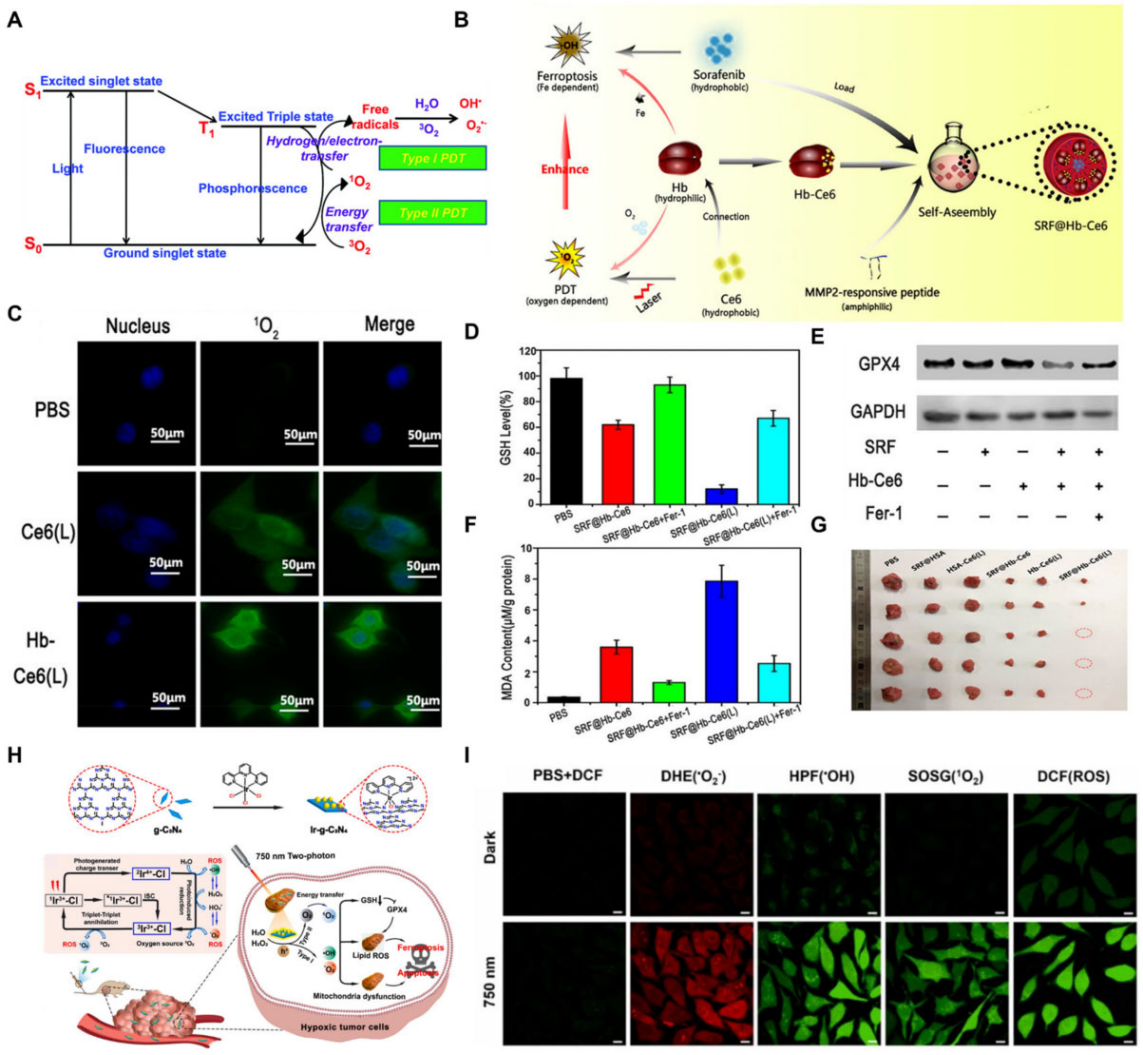
(**A**) Scheme of the photochemical reactions for type I and type II PDT. Adapted with permission from Ref. [97], © Royal Society of Chemistry 2016. (**B**) Schematic illustration displaying the oxygen-boosted phototherapy based on a 2-in-1 nanoplatform of ferrous hemoglobin to enhanced ferroptosis for tumor synergistic therapy. (**C**) CLSM images of 4T1 cells stained with DCFH-DA after different treatments. (**D**) GSH level in tumor tissue. (**E**) Western blot assay of GPX4 expression. (**F**) MDA content in tumor tissue. (**G**) Photograph of harvested tumor from mice after 14 days of different treatments, including combination therapy. (B–G) Adapted with permission from Ref. [72], © American Chemical Society 2020. (**H**) Schematic illustration of the oxygen self-sufficient nano-photosensitizer (Ir-g-C_3_N_4_) upon generation of multiple types of ROS for ferroptosis-boosted PDT under hypoxia. (**I**) Determination of the type of ROS generated upon incubation of Ir-g-C_3_N_4_. (H, I) Adapted with permission from Ref. [73], © Elsevier 2022.

PDT, a noninvasive and light-activated treatment modality, is a clinically approved therapeutic procedure that has long been used to treat various cancers [91–93]. After the local activation of photosensitizers within tumor cells under light stimulation, large amounts of ROS are generated to induce chemical damage, which leads to the death of tumor cells [94–96]. According to the different mechanisms by which photosensitizers generate ROS, PDT is divided into two types: type I and type II PDT (Fig. 3A). For type I PDT, photosensitizers generate $O_{2}^{-}$ and •OH via the interaction of triplet oxygen (^3^O_2_) and water molecule (H_2_O) with free radicals, respectively. They are formed by the light-induced excited triplet of photosensitizer involved in the electron-transfer process to react with some biological substrates. For type II PDT, photosensitizers produce ROS (mainly ^1^O_2_) through the conversion of stable ^3^O_2_ into the highly reactive ^1^O_2_ elicited by the energy transfer of the excited triplet of photosensitizers with the absorption of light [97]. Accordingly, type I PDT is more beneficial for the treatment of hypoxic tumors. Moreover, because of its strong ability to generate ROS, the study of ferroptosis based on PDT to treat tumors has gradually attracted the attention of researchers [98–100].

According to the mechanism of ROS generation by photosensitizers, the application of PDT in cancer therapy is severely limited by hypoxic tumor cells, especially in type II PDT. Therefore, the key to improving the therapeutic effect of PDT is to provide sufficient oxygen. Hemoglobin (Hb), with superior oxygen-loading characteristics and iron components, is used as an oxygen-supplying agent in PDT and as an Fe pool for ferroptosis [101]. Li *et al*. [72] constructed an SRF@Hb-Ce6 nanoplatform utilizing Hb to combine PDT with ferroptosis for tumor treatment (Fig. 3B). In this nanosystem, Ce6 generated cytotoxic ^1^O_2_ from the molecular oxygen carried by Hb through laser irradiation (Fig. 3C). Importantly, the levels of GSH and GPX4 were significantly downregulated, confirming the promotion of ferroptosis (Fig. 3D and E). Subsequently, the degree of lipid peroxidation was considerably increased through treatment with SRF@Hb-Ce6(L), and the tumors were effectively eliminated after treatment with SRF@Hb-Ce6 NPs under laser irradiation (Fig. 3F and G). Overall, with the help of Hb, ^1^O_2_ produced by type II PDT enhances ferroptosis induced by SRF, and significant tumor suppressor effects have been demonstrated *in vitro* and *in vivo*.

Considering that ferroptosis based on type I PDT is more favorable for the treatment of hypoxic tumors than type II PDT, Wei *et al*. [73] developed a mitochondria-targeting oxygen self-sufficient two-photon nano-photosensitizer, referred to as Ir-g-C_3_N_4_, which combined type I and type II two-photon PDT to boost ferroptosis. Notably, iridium complexes are ideal for subcellular mitochondrial targeting owing to their inherent lipophilicity and overall positive charge. Upon two-photon irradiation at 750 nm, Ir-g-C_3_N_4_ produced oxygen from endogenous H_2_O_2_ or H_2_O and generated therapeutic species (•$O_{2}^{-}$, •OH and ^1^O_2_) via type I and type II PDT processes, respectively (Fig. 3H and I). Various ROS generated by two-photon irradiation induce distinct ferroptosis under hypoxic conditions, displaying GSH and GPX4 downregulation.

To overcome the limitation of hypoxia in the application of PDT to induce remarkable ferroptotic effects, Lu *et al*. [102] reported an osmium-peroxo complex to generate a variety of ROS under light irradiation without molecular oxygen to trigger ferroptosis, which was distinct from the mechanism of O_2_-dependent type I or type II PDT processes. However, small-molecule drugs have intrinsic drawbacks such as poor water solubility and low cancer selectivity. Accordingly, if ferroptosis-inducing agents based on improved photosensitizers are integrated into nanotechnology in the future, they may show better ferroptosis-inducing effects and hypoxic tumor treatment effects.

#### Ultrasound stimuli-responsive nanosystems based on SDT for inducing ferroptosis

SDT, an ultrasound stimuli-responsive therapy, has been widely studied and explored as a new type of external stimulus, first proposed by Umemura *et al*. in 1989 [103]. SDT mainly uses low-intensity ultrasound to activate the sonosensitizer, which produces a large amount of cytotoxic ROS to kill tumor cells [104, 105]. Although PDT exhibits excellent performance in the clinic, the penetration depth of light in tissues seriously limits its application [95]. Fortunately, ultrasound has a high tissue-penetrating capability of up to several tens of centimeters, with minimal side effects on the surrounding normal tissues, suggesting that SDT is more beneficial for the treatment of deep-seated tumors [106]. Similar to the ROS generation mechanism in PDT, sonosensitizers absorb energy and go from the ground state to the excited state under ultrasound. When the transition electrons return to the ground state, they release a large amount of energy and act on the surrounding oxygen molecules to generate a series of ROS substances, such as ^1^O_2_ and •OH [107]. The massive intracellular ROS formation induced by SDT also induces ferroptosis. The combination of SDT and ferroptosis provides a novel strategy for cancer treatment.

**Figure 4.**
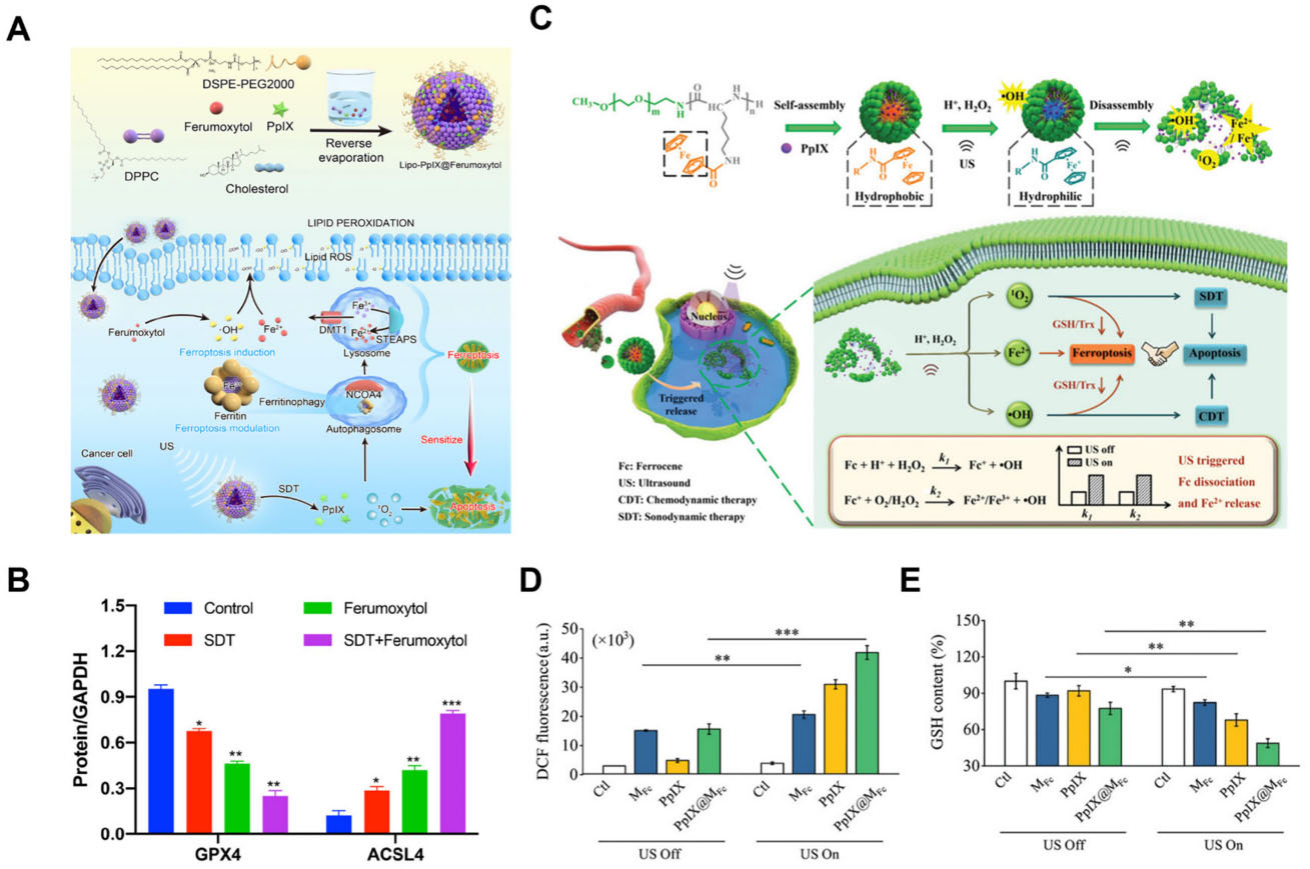
(**A**) Schematic illustration for the preparation of the engineered nanoliposomes and underlying synergistic therapeutic mechanism of SDT-based ferroptosis-targeting. (**B**) Quantitative analysis of western blot on the expression of key ferroptosis makers including GPX4 and ACSL4 after different treatments. Adapted with permission from Ref. [78], © Elsevier 2021. (**C**) Schematic illustration of the mechano-responsive leapfrog micelles (PpIX@MFc) for apoptotic and ferroptotic cancer therapy. (**D**) Generation of total ROS in formulation-incubated 4T1 cells with or without ultrasound (US) treatment (*n* = 3). (**E**) The influence of ultrasound (US) on the GSH in 4T1 cells treated by three formulations (PpIX, MFc and PpIX@MFc micelles) (*n* = 3). In (**D**) and (**E**), *P < 0.05, **P < 0.01, and ***P < 0.001. (C–E) Adapted with permission from Ref. [79], © Wiley 2022.

Here are some typical studies on ferroptosis inducers based on SDT. Zhou *et al*. [78] synthesized nanosonosensitizer (PpIX)-based nanoliposomes to achieve the anti-tumor effect of SDT combined with ferroptosis. In the Lipo-PpIX@Ferumoxytol nanosystem, ferumoxytol, a type of iron oxide NP, and PpIX, a model sonosensitizer, exhibited a ferroptosis-inducing function by elevating the intracellular ROS and lipid peroxide levels under ultrasound irradiation (Fig. 4A). The ferroptotic damage induced by Lipo-PpIX@Ferumoxytol nanoliposomes was confirmed by the reduced expression of GPX4 and higher expression level of ACSL4 in cancer cells (Fig. 4B). Using the similar mechanism, Li *et al*. [79] developed a mechano-responsive platform to trigger apoptosis and ferroptosis in cancer cells via a novel ‘leapfrog’ polymeric micelle in the following year. Ultrasound triggered the release of Fe^2+^ from ferrocene (Fc) (Fig. 4C) to react with H_2_O_2_ present in the tumor microenvironment to generate •OH. The dissociation of Fc initiated the dissemination of micelles to release PpIX, producing ^1^O_2_ with the presence of ultrasound irradiation. The upregulated ROS consumed large amounts of GSH to induce ferroptosis (Fig. 4D and E).

SDT has many advantages, such as higher tissue penetration, catalytic properties and precise spatiotemporal control. However, the therapeutic effect of SDT is severely influenced by hypoxia and high GSH levels in tumor tissues. Therefore, a sonosensitizer that has the capacity for self-sufficiency of O_2_ and GSH depletion will draw extensive attention.

**Figure 5.**
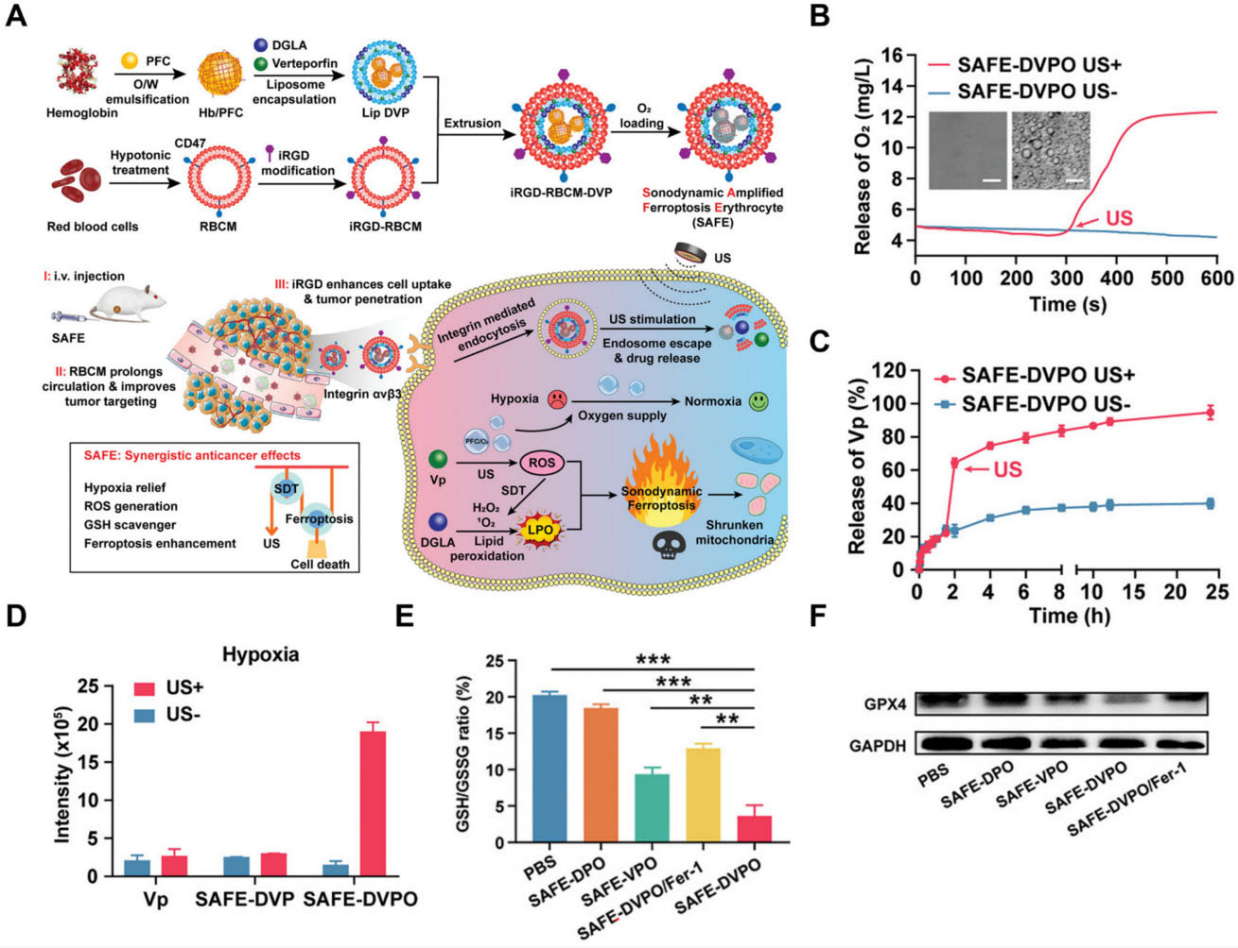
(**A**) Schematic illustration of preparation of SAFE and SAFE-mediated combination treatment of SDT and ferroptosis. (**B** and **C**) Ultrasound-triggered oxygen (B) and Vp (C) release from SAFE-DVPO. SAFE-DVPO in PBS was treated with or without ultrasound stimulation (0.35 W cm^−2^, 1 MHz). (**D**) Measurement of SAFE-DVPO mediated sonodynamic effects under hypoxia. (**E**) GSH/GSSG ratios in 4T1 cells after the different treatments, *P < 0.05, **P < 0.01, and ***P < 0.001. (**F**) The expression levels of GPX4 in 4T1 cells after the different treatments. Adapted with permission from Ref. [80], © Wiley 2022.

To satisfy this requirement, Ning *et al*. [80] synthesized an ultrasound-activated O_2_ and ROS production nanoplatform called SAFE to combine ferroptosis and SDT for hypoxic tumor therapy (Fig. 5A). In the SAFE nanoplatform, perfluorocarbon droplets acted as oxygen carriers, which were converted into microbubbles under ultrasound stimulation to rapidly release Vp and O_2_ (Fig. 5B and C). Moreover, the release of abundant oxygen effectively alleviated the hypoxic environment of tumors and was also used as a sonosensitizer to generate large amounts of ^1^O_2_ through ultrasound stimulation under hypoxia (Fig. 5D). Meanwhile, the intracellular GSH level was significantly lower after SAFE-DVPO treatment than that in the other groups (Fig. 5E). The redox imbalance caused by ROS production and GSH depletion was significantly amplified, resulting in a marked increase in the efficiency of ferroptosis, as manifested by a significant decrease in GPX4 activity (Fig. 5F). These results indicate that oxygen self-supply nanosystems may be a better choice for overcoming the limitations of SDT and ferroptosis combination for the treatment of hypoxic tumors. These studies indicate that activated sonosensitizers triggered by mechanical ultrasound generate ROS and induce ferroptosis. Further studies on anti-cancer strategies based on the combined application of SDT and ferroptosis are expected.

#### Other external stimuli-responsive nanosystems for inducing ferroptosis

Besides the abovementioned three common external stimuli-responsive nanosystems, there are some nanomaterials based on other external stimuli that induce ferroptosis, including MF and radiofrequency, which will be summarized and illustrated by some specific examples in this section.

Owing to the minimum physical interaction with the human body, considerable attention has been paid to developing external stimuli-responsive nanosystems based on an MF, which is considered a better choice than other common external stimuli [108]. Yu *et al*. [83] developed ascorbic acid (AA) and iron oxide nanocubes (IONCs)-encapsulated poly (lactic-co-glycolic acid) vesicles, which used MF to promote ferroptosis-like cell death. In external circularly polarized MF, AA was released to reduce the ferric ions in the IONCs to ferrous ions to induce the Fenton reaction, successfully boosting external MF-triggered ferroptosis-like cell death. In addition, Yang *et al*. [109] developed lipoxidase nanoreactors incorporating radiofrequency ablation (RFA) therapy to induce ferroptosis. Under the action of RFA, massive tumor debris was generated to provide PUFA for lipoxidase-triggered lipid peroxidation, thereby amplifying the power of ferroptosis.

Collectively, these nanomaterials provide a laboratory of nanosystems for ferroptosis under different external stimuli to disrupt intracellular redox homeostasis, ultimately inducing ferroptosis for cancer therapy. These types of stimuli-responsive therapies have made outstanding contributions to the development of novel tumor therapy methods by inducing ferroptosis. However, many questions need to be resolved before their clinical application, such as the dosage transformation from mice to patients and the power of the external-stimuli generator.

### Internal stimuli-responsive nanosystems for inducing ferroptosis

The specificity of the tumor microenvironment provides multiple targets for designing internal stimuli-responsive nanosystems to induce ferroptosis, including ROS, GSH, pH and glucose. Generally, the amount of ROS and GSH are higher in cancer cells than in normal cells [110, 111], making them the prospective targets for designing drug delivery systems. Besides, the acidic microenvironment distinguishes tumor tissue from healthy tissue, rendering it one of the most popular responsive features to develop novel nanocarriers [112]. Moreover, the oxidation of intracellular glucose can generate H_2_O_2_ to increase ROS levels, thereby promoting ferroptosis [113]. Therefore, glucose is an effective target for internal stimulus-responsive nanosystems. These unique internal stimuli of tumor cells open new avenues for designing novel tumor therapeutic nanosystems via ferroptosis. This section summarizes different internal stimuli-based responsive nanomaterials for ferroptosis. Representative internal stimuli-responsive nanosystems used to induce ferroptosis are listed in Table 2.

**Table 2.** The representative internal stimuli-responsive nanosystems used for inducing ferroptosis

Internal stimulus	Nanosystems	Responsive composition	Core mechanism	*In vitro*/*in vivo* model	Ref
ROS	siMCT4-PAMAM-PEG-TK-Fc@DEM	Thioketal groups	ROS accumulation, GSH depletion, GPX4 downregulation and lipid peroxidation	4T1 cells/BALB/c mice	[114]
	PCFD	Thioketal groups	ROS accumulation, GSH depletion, GPX4 downregulation and lipid peroxidation	4T1 cells/BALB/c mice	[115]
	PTAF	Thioacetal groups	ROS accumulation, GSH depletion, GPX4 downregulation and lipid peroxidation	4T1 cells/BALB/c mice	[116]
	CM-DSe-SRF-Fe^2+^	Diselenide bond	ROS accumulation, GSH depletion, GPX4 downregulation and lipid peroxidation	A549 cells/BALB/c mice	[117]
GSH	HL/MOS@M780&LOD	Disulfide bonds	ROS accumulation, GSH depletion, GPX4 downregulation and lipid peroxidation	4T1 cells/BALB/c mice	[118]
	FCS/GCS	Disulfide bonds	ROS accumulation, GSH depletion, GPX4 downregulation and lipid peroxidation	4T1 cells/BALB/c mice	[119]
	MMSNs@SO	Manganese-oxygen bonds	GSH depletion, inhibition system Xc^-^, GPX4 downregulation and lipid peroxidation	HepG2 cells and H22 cells/No studies in vivo	[120]
	CDC@SRF	α, β-unsaturated ketones	GSH depletion, inhibition system Xc^-^, GPX4 downregulation and lipid peroxidation	4T1 cells/BALB/c mice	[121]
pH	BNP@R	Phenylboronate ester bonds	GSH depletion, inhibition system Xc^-^, GPX4 downregulation and lipid peroxidation	4T1 cells/BALB/c mice	[122]
	cPFC_DBCO_/cPFC_N3_	Maleic acid amide/poly (2-azepane ethyl methacrylate) (PAEMA)	ROS accumulation, GSH depletion, GPX4 downregulation and lipid peroxidation	4T1 cells/BALB/c mice	[123]
	UCNP@GA-Fe^III^	Fe-GA complexes	ROS accumulation	LS180 cells/BALB/c mice	[124]
	DOX/Fe^3+^/EGCG NPs	Fe-EGCG complexes	ROS accumulation, GPX4 downregulation and lipid peroxidation	LL2 cells/A549/C57 mice	[125]
	SR780@Fe-PAE-GP	Fe-SR780	ROS accumulation, GSH depletion, GPX4 downregulation and lipid peroxidation	HepG2 cells/BALB/c mice	[126]
	FMMHG/Sor	Fe_3_O_4_/MnO_2_	ROS accumulation, GSH depletion, GPX4 downregulation and lipid peroxidation	A549 cells/BALB/c mice	[127]
Glucose	NMIL-100@GOx@C	GOx	ROS accumulation, GSH depletion, GPX4 downregulation and lipid peroxidation	4T1 cells/BALB/c mice	[128]
	Au/Cu-TCPP(Fe)@RSL3 -PEG-iRGD	Au NPs	ROS accumulation, GSH depletion, GPX4 downregulation, lipid peroxidation and inhibition CoQ10H2 synthesis	4T1 cells/BALB/c mice	[129]

#### ROS-responsive nanosystems for inducing ferroptosis

Given the significant differences in ROS levels between normal and tumor tissues [130, 131], some ROS-responsive nanosystems of ferroptosis inducers have gradually been developed for tumor therapy by introducing ROS-sensitive chemical bonds [132, 133]. Specifically, ROS-responsive delivery systems induce ferroptosis by loading small-molecule inducers of ferroptosis, which improves their aqueous solubility and targeting properties, enabling precise drug release in the tumor environment [114]. Furthermore, genes related to ferroptosis are also integrated into nanosystems by introducing ROS-responsive moieties to improve their metabolic stability and biocompatibility [29]. In this section, ROS-responsive ferroptosis inducers are classified according to ROS-responsive groups, such as thioketal and selenium-based groups.

##### Thioketal groups

Thioketal groups are one of the most commonly used groups in ROS-responsive nanosystems. The specific response mechanism of delivery systems based on thioketal groups is the chemical bond cleavage of thioketals in an H_2_O_2_ environment to generate ketones and thiols [134], leading to the subsequent degradation of NPs to release the loaded drug. ROS-responsive delivery agents based on thioketal groups have been extensively explored and reported in recent years [135, 136]. Below are some examples of recently reported ROS-responsive delivery systems based on thioketal groups that induce ferroptosis.

**Figure 6.**
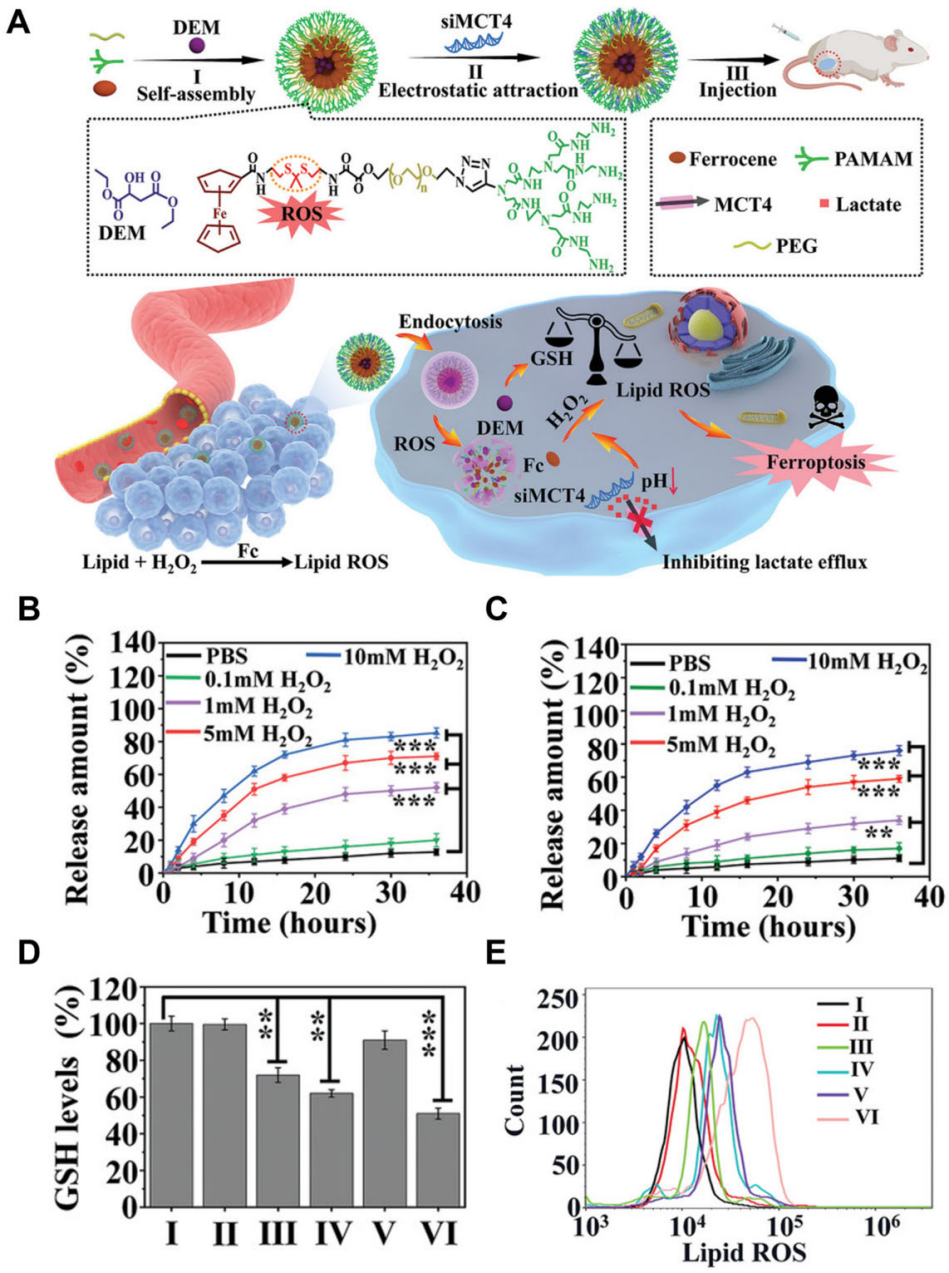
(**A**) Schematic illustration of preparation process for siMCT4-PAMAM-PEG-TK-Fc@DEM and the underlying mechanism for ferroptosis-based tumor therapy. (**B** and **C**) DEM and siMCT4 release from nanoassembly under redox conditions (0.1, 1, 5, 10 × 10^−3^ mM H_2_O_2_). (**D**) Changes in relative intracellular GSH levels of 4T1 cells after various treatment for 24 h (Control (I), PAMAM-PEG-TK-Fc (II), DEM (III), PAMAM-PEG-TK-Fc@DEM (IV), siMCT4-PAMAM-PEG-TK-Fc (V) and siMCT4-PAMAM-PEG-TK-Fc-DEM (VI)). (**E**) Flow cytometric analysis on the intracellular lipoperoxide levels in 4T1 cells incubated with Control (I), PAMAM-PEG-TK-Fc (II), DEM (III), PAMAM-PEG-TK-Fc@DEM (IV), siMCT4-PAMAM-PEG-TK-Fc (V) and siMCT4PAMAM-PEG-TK-Fc-DEM (VI) for 24 h. In (**B**), (**C**) and (**D**), *P < 0.05, **P < 0.01, and ***P < 0.001. Adapted with permission from Ref. [114], © Wiley 2022.

For instance, Zhang *et al*. [114] developed a ferroptosis-inducing agent by incorporating thioketal groups as oxidation-responsive functional groups. In this study, the thioketal linkage was the key factor in designing the ROS-responsive nano-assembled platform. This platform rapidly disassembled due to the ROS-mediated fracture of the thioketal linkage after entering the tumor tissue and released the incorporated therapeutic components (Fig. 6A). Large amounts of diethyl maleate (DEM) and monocarboxylate transporter 4-inhibiting siRNA (siMCT4) were released after treatment with 10 × 10^−3^ M H_2_O_2_. However, their release percentages were negligible in the absence of H_2_O_2_ (Fig. 6B and C), suggesting that the nanoassembly was stable under normal physiological conditions, and H_2_O_2_ triggered drug release by breaking the thioketal bond. DEM-induced GSH reduction due to thioketal bond cleavage and siMCT4-mediated ROS production amplified the damage induced by ferroptosis (Fig. 6D and E). In this study, the researchers designed an on-demand drug release nanoplatform using an ROS-responsive thioketal bond to trigger ferroptosis and enhance anti-tumor efficacy.

**Figure 7.**
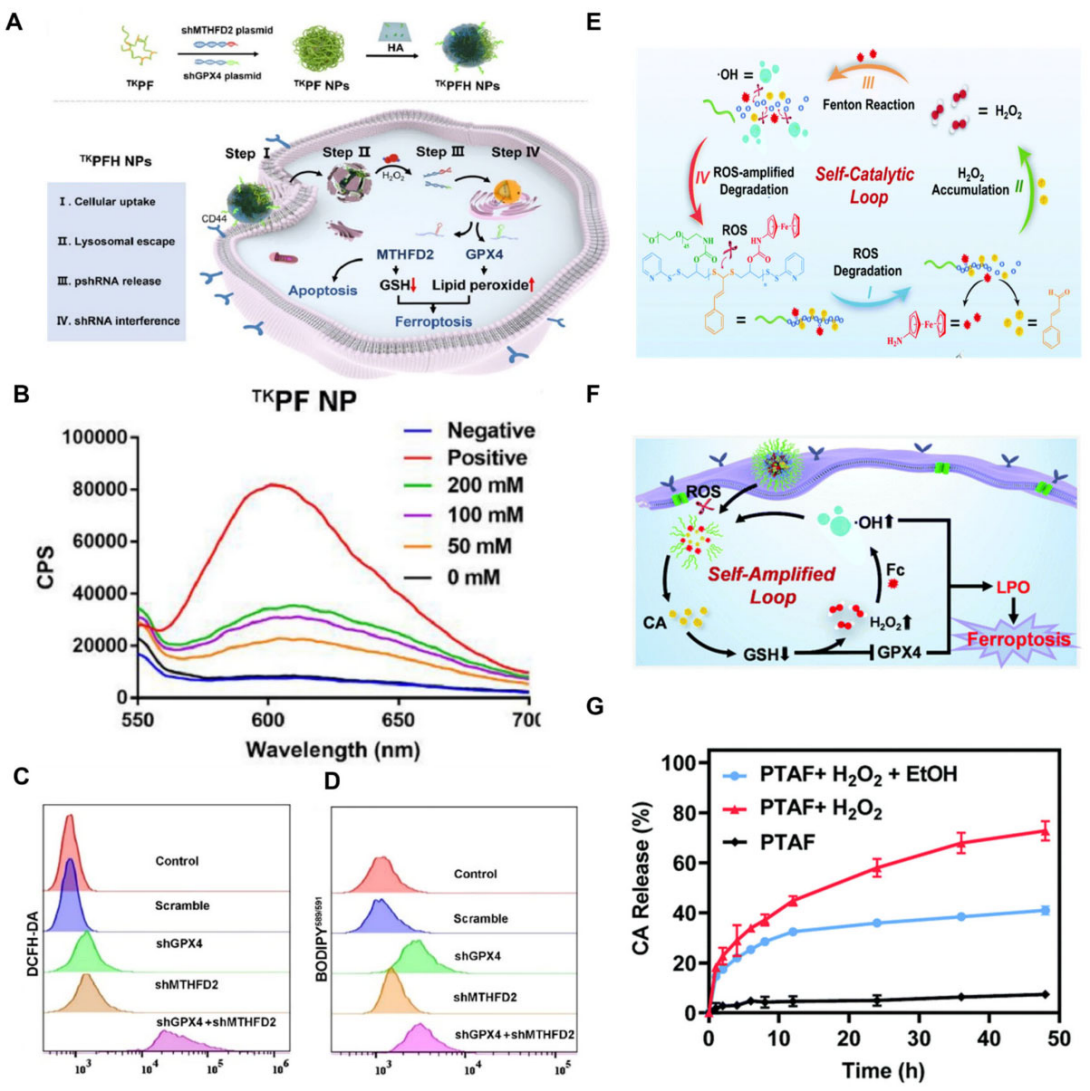
(**A**) Schematic illustration of the fabrication processes of ^TK^PFH NPs and anticancer mechanisms of triggering apoptosis and ferroptosis for cancer therapy. (**B**) Fluorescence emission of ^TK^PFH encapsulating EB-pDNA treated with different amount of H_2_O_2_ (0–200 mM); spectroscopy ranging from 550 nm to 700 nm. (**C**, **D**) ROS generation (C) and lipid peroxides (D) detection of cells transfected by ^TK^PFH encapsulating scramble, shGPX4, shMTHFD2 and shGPX4 + shMTHFD2. (Scale bar: 100 μm.) Adapted with permission from Ref. [29], © Elsevier 2022. (**E**) The establishment process of the ROS self-catalytic loop triggered by TA-Fc-PEG in response to ROS. (**F**) Mechanistic evaluation of PTAF-induced ferroptosis via ROS self-catalytic loop. (**G**) CA release profiles of PTAF under different treatments. (E–G) Adapted with permission from Ref. [116], © Royal Society of Chemistry 2022.

A similar work was reported by Yang *et al*. [29], wherein the researchers developed an ROS-responsive gene vector to deliver shMTHFD2 and shGPX4 plasmids to suppress the expression of MTHFD2 and GPX4 to trigger ferroptosis and apoptosis. The gene vector ^TK^PFH NP platform was fabricated using thioketal-crosslinked fluorinated polyethyleneimine (^TK^PF) coated with hyaluronic acid (Fig. 7A). ^TK^PF NPs rapidly disintegrated to release shMTHFD2 and shGPX4 plasmids due to the reaction of the thioketal group with abundant ROS (Fig. 7B). The ferroptosis effect was enhanced by the elevated levels of ROS and lipid peroxides triggered by shMTHFD2 and shGPX4 plasmids and disruption of intracellular redox homeostasis (Fig. 7C and D).

In addition, thioacetal, a group similar to thioketal, has been used to design ROS-responsive ferroptosis inducers. Li *et al*. [116] prepared a ferroptosis self-catalyst called PTAF using Fc conjugated with a cinnamaldehyde (CA)-containing polymer chain with a thioacetal group (Fig. 7E). With ROS-triggered thioacetal bond cleavage, PTAF dissociated, followed by the release of 70% CA, leading to H_2_O_2_ accumulation to induce the Fenton reaction, which generated •OH and ultimately triggered ferroptosis (Fig. 7F and G).

ROS-responsive delivery systems based on thioketal groups have been used for precise drug release and contribute to the induction of ferroptosis in cancer therapy. Notably, the delivery system modified by the thioketal groups accelerated the release of the drug at higher H_2_O_2_ concentrations in the tumor environment. ROS-responsive ferroptosis inducers based on thioketal groups are rare, and may be a promising research direction.

##### Selenium-based groups

Selenium-based groups are a class of classical groups that exhibit redox sensitivity. Unlike the abovementioned thioketal groups, selenium-based groups respond not only to ROS but also to GSH [137]. This dual-response feature makes it a promising moiety for drug delivery. Selenium-based groups come in two forms: monoselenide and diselenide groups. In the presence of H_2_O_2_, the responsive mechanism of delivery systems based on monoselenide or diselenide groups is that they transform to selenium sulfone or selenic acid [52], leading to the subsequent degradation of NPs and drug release. Taking advantage of this redox-mediated transition, a redox-sensitive ferroptosis inducer has been synthesized.

**Figure 8.**
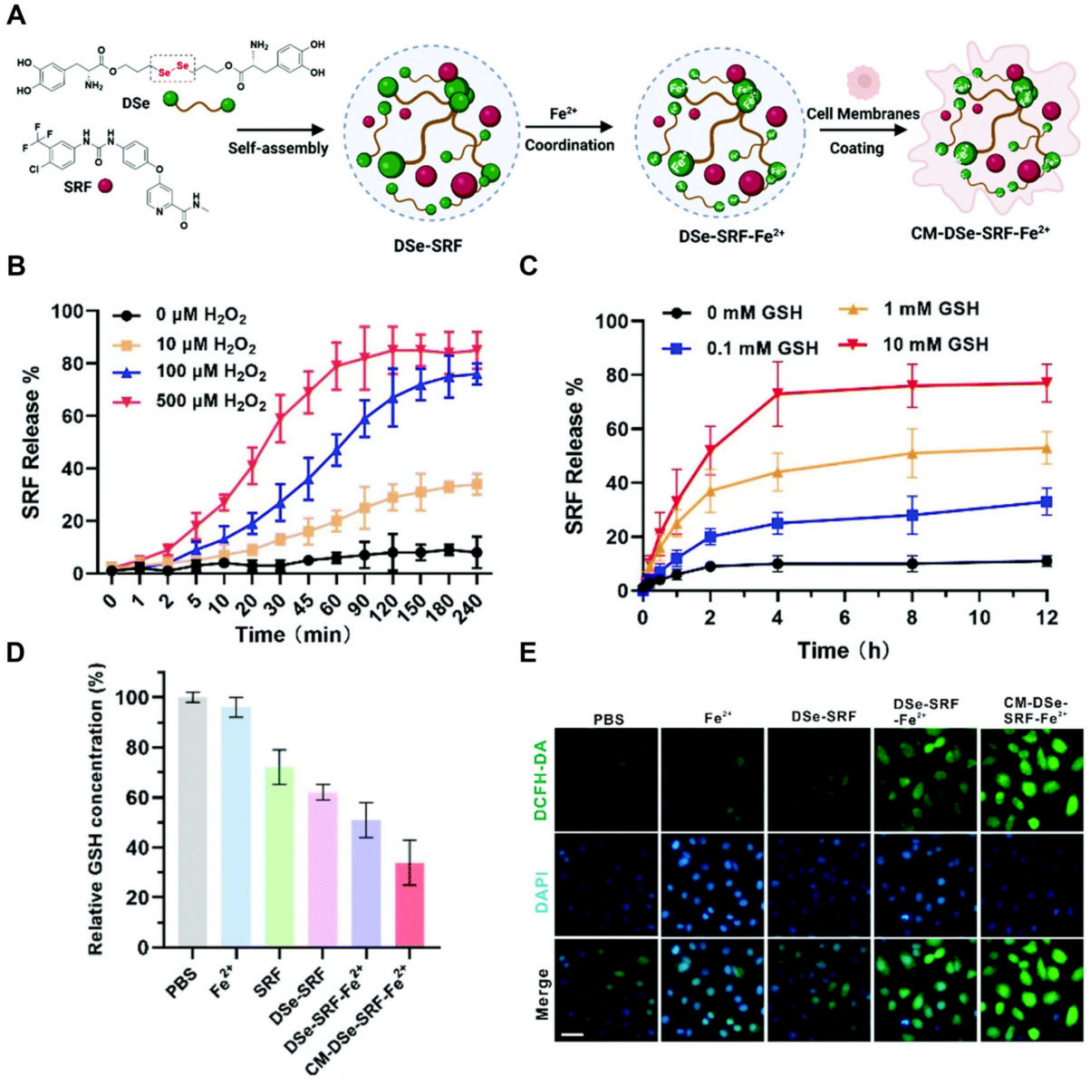
(**A**) Schematic illustration for the preparation of the CM-DSe-SRF-Fe^2+^. (**B**) SRF release behavior from DSe-SRF-Fe^2+^ in the presence of H_2_O_2_ (0, 10, 100 and 500 μM) for 240 min (pH 7.4 PBS buffer at 37°C). (**C**) SRF release behavior from DSe-SRF-Fe^2+^ in the presence of GSH (0, 0.1, 1 and 10 mM) for 12 h (pH 7.4 PBS buffer at 37°C). (**D**) GSH level of different formulations (PBS, Fe^2+^, DSe-SRF, DSe-SRF-Fe^2+^ and CM-DSe-SRF-Fe^2+^) in A549 cells. (**E**) ROS generation of different formulations (PBS, Fe^2+^, DSe-SRF, DSe-SRF-Fe^2+^ and CM-DSe-SRF-Fe^2+^) was determined using the ROS Assay Kit, and the green fluorescence was from DCFH-DA (ROS indicator) and the blue fluorescence was from DAPI (nuclei); scale bar: 50 μM. Adapted with permission from Ref. [117], © Royal Society of Chemistry 2022.

Ping *et al*. [117] reported an oxidative stress stimuli-responsive self-assembled nanodrug based on a dynamic diselenide bond to enhance ferroptosis. Diselenide-bridged levodopa (DSe) connected to SRF to self-assemble into a dually functional NP (DSe–SRF), followed by chelation with Fe^2+^ to form the nanodrug DSe–SRF–Fe^2+^, followed by A549 cell membrane coated onto DSe–SRF–Fe^2+^ to form CM–DSe–SRF–Fe^2+^ to enhance tumor targeting ability and boost *in vivo* stability (Fig. 8A). The diselenide bond was broken in response to abundant ROS/GSH in tumor cells, and free SRF was quickly released (Fig. 8B and C) to inhibit the synthesis of GSH (Fig. 8D) to promote ferroptosis. Meanwhile, Fe^2+^ released from the nanodrug accelerated the Fenton reaction to produce •OH, which increased the level of intracellular ROS and induced ferroptosis (Fig. 8E).

According to the abovementioned research data, the drug delivery systems based on diselenide bonds were observed to realize the drugs release in a short time under the stimulation of ROS or GSH, which provides a guarantee for the ferroptosis-inducing effect and anti-cancer activity. However, ROS-responsive ferroptosis inducers based on selenium groups have not yet been widely developed, and this strategy is expected to receive extensive attention and research.

##### Others

In addition to the aforementioned thioketal groups and selenium-based groups, there are many ROS-responsive functional groups, such as sulfide groups, thioether groups and tellurium-based groups [52]. Sulfide groups were the first to be used in an ROS-responsive delivery system. The response mechanism of sulfide groups is the oxidation-triggered conversion of hydrophobic sulfide groups into hydrophilic sulfoxides and sulfones [138]. Using the same mechanism as sulfides, thioether groups are converted into sulfoxides and/or sulfones under the action of oxidative factors [139].

ROS-responsive delivery agents based on these groups are extensively studied and reported in recent years. Therapeutic prodrugs, imaging probes and drug delivery systems based on ROS-responsive groups have been used for cancer theranostics. However, their application in the induction of ferroptosis is rare. More responsive drug delivery systems for inducing ferroptosis based on these ROS-sensitive groups need to be developed.

#### GSH-responsive nanosystems for inducing ferroptosis

Tumor cells have high levels of oxidative stress, which means that there are high levels of ROS and GSH in the cells [140]. Thus, GSH is another target for internal stimulus-responsive nanosystems. Moreover, GSH acts as a hinderance to the induction of ferroptosis because it is involved in ROS scavenging [141]. Hence, GSH-responsive nanosystems not only achieve precise drug release but also enhance the effect of ferroptosis by depleting GSH to break the redox balance of tumor cells [125]. According to the existing research, GSH-sensitive chemical bonds that induce ferroptosis have disulfide bonds (-S-S-) [142], diselenide bonds (-Se-Se-) [117], and manganese–oxygen bonds (-Mn-O-) [143]. In this section, GSH-responsive ferroptosis inducers are classified according to their GSH-responsive chemical bonds.

##### Disulfide bonds

As the most common GSH-sensitive chemical bond, disulfide bonds are involved in the design and synthesis of numerous GSH-responsive nanoplatforms [144–147]. Disulfide bonds generate sulfhydryl groups through a thiol–disulfide exchange reaction with the thiols of GSH, causing irreversible cleavage [148]. Given that the depletion of GSH plays a key role in the induction of ferroptosis, it has also been widely used in the design of ferroptosis inducers.

**Figure 9.**
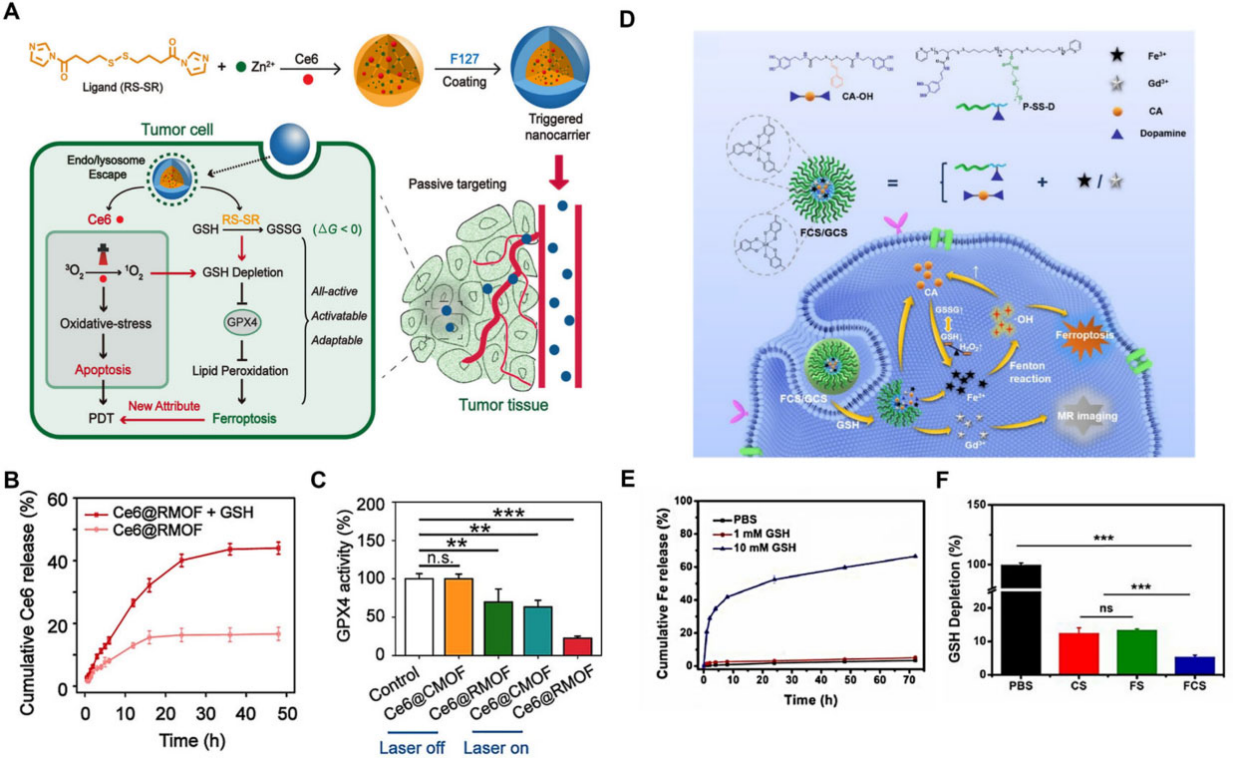
(**A**) Schematic illustration of all-active metal organic framework nanocarriers for antitumor PDT that involves both apoptosis and ferroptosis. (**B**) Cumulative Ce6 release from Ce6@RMOF with or without 10 mM GSH treatment (*n* = 3). (**C**) Activity of GPX4 in 4T1 cells upon incubation with Ce6@CMOF or Ce6@RMOF nanocarriers, *P < 0.05, **P < 0.01, and ***P < 0.001. Adapted with permission from Ref. [142], © American Chemical Society 2019. (**D**) Schematic illustration of the processes for cancer imaging and therapy. (**E**) Cumulative release of Fe from FCS. (**F**) GSH depletion capacity of CS, FS and FCS, *P < 0.05, **P < 0.01, and ***P < 0.001. (D–F) Adapted with permission from Ref. [119], © Elsevier 2022.

For instance, Meng *et al*. [142] employed a redox-responsive MOF nanocarrier (Ce6@RMOF) to achieve anti-tumor effects based on ferroptosis and PDT (Fig. 9A). In this MOF nanocarrier, the imidazole moieties linked by disulfide bonds coordinated with zinc to form the RMOF, which was disassembled in a high-GSH environment via a thiol–disulfide exchange reaction, causing GSH depletion and the release of Ce6 (Fig. 9B). The generation of singlet oxygen by Ce6 under light conditions exacerbated the consumption of GSH, resulting in the inactivation of GPX4 and eventual significant ferroptosis for cancer therapy (Fig. 9C). Similarly, Luo *et al*. [119] fabricated a disulfide-based GSH-responsive nanoplatform (FCS/GCS) to amplify oxidative stress for tumor ferroptosis (Fig. 9D). Specifically, the researchers incorporated iron ions (Fe^3+^)/gadolinium ions (Gd^3+^), amphiphilic polymer skeletal (P-SS-D) containing disulfide bonds and phenolic hydroxyl groups, and a cinnamaldehyde prodrug (CA-OH) via chelation reactions between metals and polyphenols to form the FCS/GCS nanosystem. The NPs were depolymerized by cleavage of disulfide bonds in the high-GSH tumor microenvironment to release Fe^3+^ to catalyze the Fenton reaction to generate •OH (Fig. 9E). The depletion of GSH and production of •OH amplified oxidative stress in tumor cells, ultimately inducing ferroptosis for cancer therapy (Fig. 9F).

##### Others

In addition to the abovementioned disulfide bonds, which have been reported for the design of GSH-responsive ferroptosis inducers, several other chemical bonds have also been used to design such nanoplatforms, such as diselenide bonds, manganese–oxygen bonds and α, β-unsaturated ketones.

The diselenide bond, one of the GSH-responsive chemical bonds, is cleaved to generate hydrophilic selenol under the action of GSH [149]. To date, there have been many reports on the design of stimuli-responsive nanomaterials based on diselenide bonds [150–152]. However, there have been very few studies on GSH-responsive nanomaterials based on diselenide bonds to induce ferroptosis and the only one example searched by us is detailed in reference [117].

**Figure 10.**
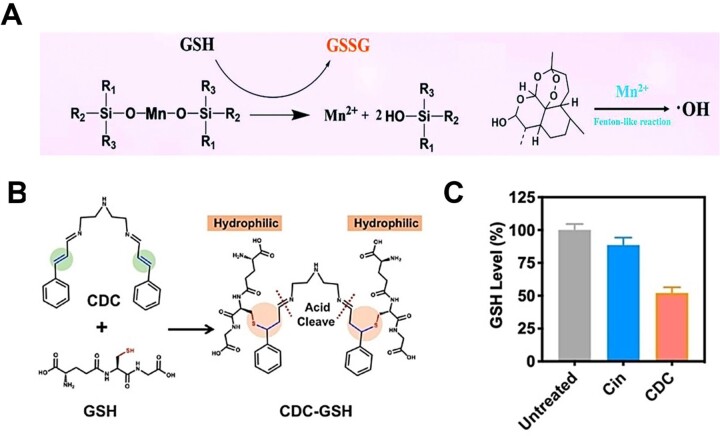
(**A**) Chemical reaction equations of GSH-exhaustion and •OH production by FaPEG-MMSNs@DHA in tumor cells. Adapted with permission from Ref. [143], © Royal Society of Chemistry 2020. (**B**) Molecular mechanism of pH/GSH responsive disassembly of CDC dimersomes. (**C**) Intracellular GSH level after incubation with Cin or CDC (*n* = 3). (B, C) Adapted with permission from Ref. [121], © Wiley 2022.

Manganese–oxygen bonds, decomposed in the reducing microenvironment, are broken under the action of two molecules of GSH to generate one molecule of Mn^2+^ and two molecules of the corresponding alcohol. More importantly, ferroptosis is aggravated by the formation of •OH via a Fenton-like reaction between Mn^2+^ and H_2_O_2_ (Fig. 10A). Based on the GSH-responsive properties, the researchers designed the manganese-doped mesoporous silica NPs (syncopated as MMSNs), which were synthesized via introducing manganese–oxygen bonds (-Mn-O-) into the framework of ordinary mesoporous silica NPs (-Si-O-Si-). Tang *et al*. [120] utilized MMSNs as carriers to load SRF to induce ferroptosis in tumor cells. In addition, MMSNs were used to carry dihydroartemisinin for ferroptotic cancer therapy in a study by Fei *et al*. [143]. In these two reports, manganese–oxygen bonds exhibited excellent GSH-responsive drug release and GSH-depletion capabilities.

α, β-unsaturated ketones react with the thiol group of GSH via Michael addition to realize the response and consumption of GSH. Inspired by this, Zhou *et al*. [121] prepared amphiphilic lipid-like cinnamaldehyde (containing α, β-unsaturated ketones) dimers (CDC) to encapsulate SRF to significantly enhance ferroptosis. Binding of hydrophilic GSH to CDC disrupted the amphipathic nature of the lipid-like structure, triggering disassembly of the CDC dimer (Fig. 10B), enabling GSH-responsive drug release and GSH depletion, and facilitating ferroptosis (Fig. 10C).

The GSH depletion properties of GSH-responsive chemical bonds have a non-negligible advantage and prospects for inducing ferroptosis. The introduction of GSH-responsive chemical bonds into nanocarriers improves tumor targeting while simultaneously consuming intracellular antioxidant GSH to disrupt the redox balance in tumor cells, which is the key factor leading to ferroptosis. Overall, GSH serves as a target for ferroptosis-related drug-responsive release to enhance ferroptosis in cancer therapy. There are several types of GSH-responsive chemical bonds, but they have not been widely used to design ferroptosis inducers. It is hoped that more ferroptosis inducers based on GSH-responsive chemical bonds will be reported in the future.

#### pH-responsive nanosystems for inducing ferroptosis

The main energy of tumor cells comes from oxygen-independent glycolysis, which produces large amounts of protons and carbon dioxide [153]. To maintain normal functions and avoid acidosis, tumor cells upregulate the carbonic anhydrase enzymes on their surface to transport those metabolic substances to the microenvironment [154]. However, the rapid growth of tumor cells makes most of them far from the blood vessel, leading to the clear of metabolic waste in the microenvironment untimely. Therefore, many acidic wastes accumulate in this area to make the pH value here (pH 6.5–6.8) lowering than that of the healthy tissue (pH 7.4) [155]. This unique feature of tumor has inspired the development of various tumor-targeting nanosystems with smart-responsive properties by containing the pH stimuli-responsive composition, such as pH-stimulated chemical structure transformation, including the charge changes or bond cleavage [156, 157]; acid-responsive nanostructure decomposition, including metal-based nanocomplexes [158]. Those advantages facilitate the delivery of small ferroptosis inducers with more safety and efficiency. What is more important, the release of transition metal elements from the metal-based nanocomplexes own the ability to catalyze H_2_O_2_ to high toxic •OH and boost ferroptosis [159, 160]. Here, we chose phenylboronate ester bonds (PBE) and metal–polyphenol complexes as representative examples to illustrate the application of those acid-responsive nanosystems induced ferroptosis in tumor therapy.

##### Phenylboronate ester bonds

Phenylboronate acid (PBA) and its derivatives are capable of forming the reversible covalent complex with the compounds containing vicinal diol structure, widely used in the delivery of insulin [161]. However, it’s hard for researchers to use PBA and its derivatives as the pH-responsive group for their high dissociation constant value (pKa 7.8–8.6). The exploration of PBE bond between PBA and 1, 2-diol (or 1, 3-diol) greatly remedies this defect. PBE bond is stable in the physiological environment (pH 7.4) and can be broken with the presence of weak acid [162]. The sensitivity of the PBE bond against acidic conditions makes it possible to develop diverse pH-responsive drug delivery systems based on this function group in the field of tumor therapy, including the delivery of ferroptosis inducers with small molecular weight.

**Figure 11.**
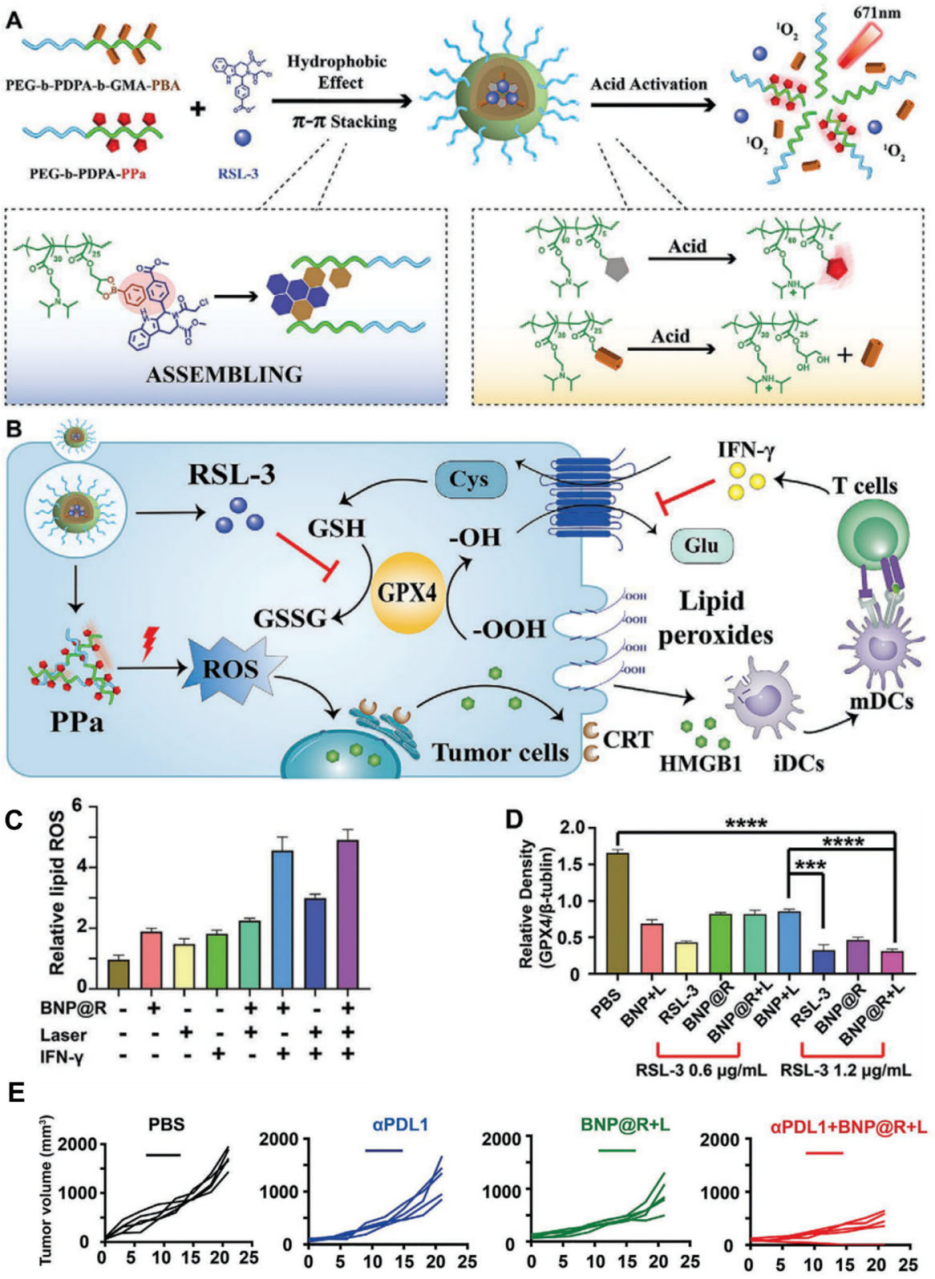
(**A**) Schematic illustration of fabrication of the acidity-activatable dynamic nanoparticles for co-encapsulating GPX4 inhibitor RSL-3. (**B**) Schematic illustration of acidity-activatable nanoparticles for improving cancer immunotherapy. (**C**) Flow cytometric analysis of PDT and GPX4 inhibition-induced intracellular accumulation of lipid peroxide in B16-F10 tumor cells *in vitro* by BODIPY-C11 fluorescent probe. (**D**) Semi-quantitative analysis of GPX4 expression in the B16-F10 tumor cells upon 24 h incubation with the BNP@R nanoparticles, *P < 0.05, **P < 0.01, and ***P < 0.001. (**E**) Tumor growth of 4T1-tumor-bearing BALB/c mice with different treatments (*n* = 5). Adapted with permission from Ref. [122], © Wiley 2021.

For example, Song *et al*. [122] recently designed an intracellular-acidity-activatable decomposition nanosystem (BNP) based on PBE bond to delivery RSL-3, a GPX4 inhibitor, to enhance drug utilization and bypass the related side effects. Via the π-π stacking effect, the benzene ring of BNP greatly improved the drug loading capability of RSL-3 and avoided drug leakage before arriving at the target location (Fig. 11A). When BNP@R reached the endocytic vesicles (pH 5.8–6.2) of tumor cells, PBE bond could be broken to accelerate the release of RSL-3, which inhibits the expression of GPX4, aggravates lipid peroxides and induces ferroptosis (Fig. 11B–D). Lastly, the authors demonstrated that the combination of ferroptosis effect and the PDT therapy significantly ameliorated the immunosuppression microenvironment and strengthened the therapeutic effect of programmed death ligand 1 blocking treatment (Fig. 11B and E).

Because ferroptosis has been emergence just about a decade, numerous pH-responsive chemical bonds have not yet been applied in this field, such as the benzoic imine bonds, orthoester bonds, hydrazone bonds and imidazole ring. What’s more exciting, the various types and multiple response ranges of those acid-triggered function groups provide the diversity possible for the design of smart nanosystems to induce ferroptosis [163]. Therefore, this may be a promising research direction in the future.

##### Metal–polyphenol complexes

Since Frank *et al*. [164] discovered the pH-triggered self-assembly and disassembly behavior of Fe^3+^ ions and tannic acid (TA) complexes in 2013, a series of similar structures via the coordination of other metal ions and polyphenols have been rapidly developed with the acid-responsive property [165]. This structure has been widely used in various fields for the convenient synthesis process, mild reaction conditions and super sensitivity against different acidic conditions [166]. At the same time, selecting appropriate metal ions can hugely enhance the ROS production of tumor cells to drive ferroptosis. Therefore, metal–polyphenol complexes have frequently been used by researchers to regulate ferroptosis in cancer therapy.

**Figure 12.**
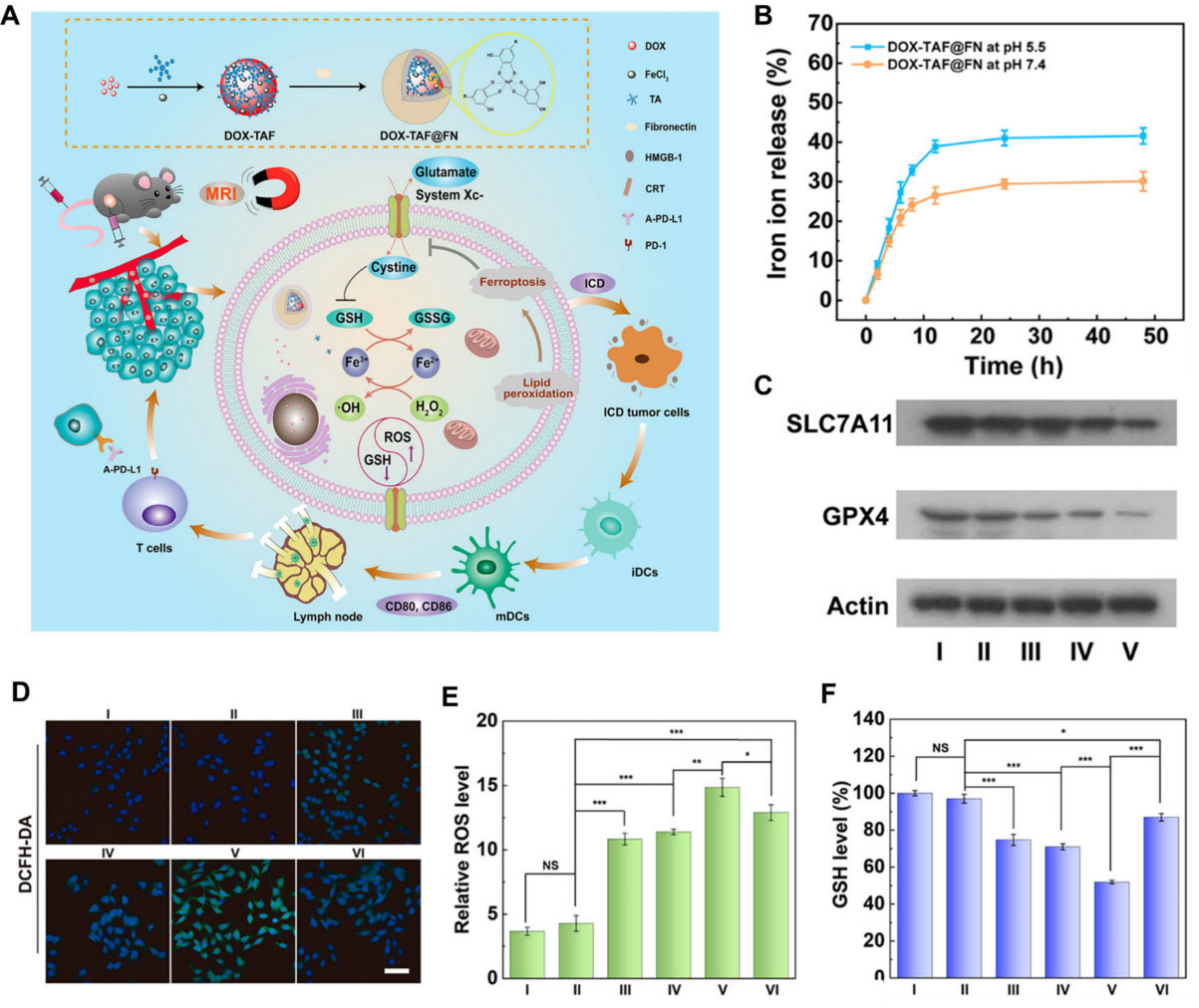
(**A**) Formation of DOX-TAF@FN nanocomplexes for *in vivo* MR imaging and combined chemo-/chemodynamic/immune therapy of tumors. (**B**) Cumulative Fe release from DOX-TAF@FN at pH 5.5 or 7.4. (**C**) Western blot analysis of the expression level of SLC7A11 and GPX4 after different treatments for 24 h. (**D**) CLSM images of B16 cells stained with DCFH-DA after different treatments for 6 h. (**E**) Relative ROS level evaluated by flow cytometry after different treatments for 6 h (*n* = 3). (**F**) GSH level in B16 cells after different treatments for 6 h (*n* = 3). GSH level in B16 cells after different treatments for 6 h (*n* = 3). In (**E**) and (**F**), *P < 0.05, **P < 0.01, and ***P < 0.001. Adapted with permission from Ref. [167], ©American Chemical Society 2022.

For example, Shi *et al*. [167] took advantage of fibronectin (FN)-coated metal–phenolic networks to induce ferroptosis and chemotherapy for tumor combination therapy. In this study, the researchers prepared FN-coated DOX-TAF nanocomposites by coordinating TA and Fe^3+^ to load DOX (Fig. 12A). The iron ions released from these nanocomposites exhibited the pH-dependent behavior (Fig. 12B). Meanwhile, DOX-TAF@FN greatly inhibited the expression of GPX4 (Fig. 12C) via the depletion of GSH (Fig. 12F) and induced a significant increase in intracellular ROS levels through the Fenton reaction with iron ions (Fig. 12D and E). This strategy showed a robust tumor inhibitory effect owing to the combination of ferroptosis, chemotherapy and immunotherapy.

**Figure 13.**
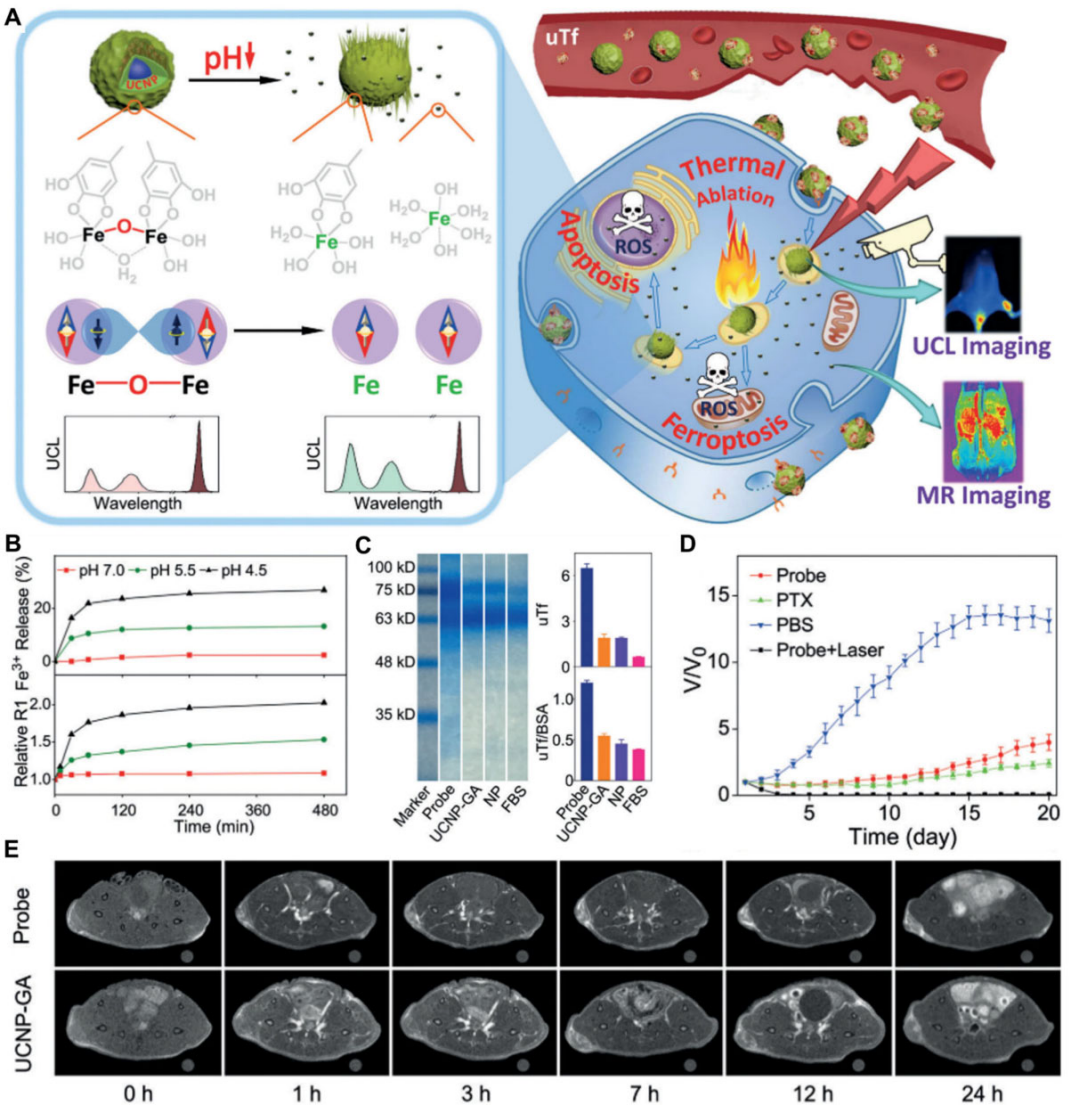
(**A**) Illustration to demonstrate the activatable function of UCNP@GA-FeIII probe for MRI and its therapeutic function involving multiple pathways. (**B**) The release kinetics of Fe^3+^ for comparing with the temporal R1 of the UCNP@GA-Fe^III^ probes recorded at different pH values on a 3.0 T MRI scanner. (**C**) Gel electrophoresis of proteins adsorbed on the probe and it control particles with quantitative data showing in the right-top frame and specific binding of uTf (defined as the ratio of uTf/BSA) showing in the right-bottom frame. (**D**) The tumor growth curves of mice bearing LS180 tumors receiving different treatments. (**E**) MR and upconversion luminescence imaging of tumors *in vivo*. T1-weighted MR images of tumor-bearing mice acquired at different time points pre- and post-injection of the probe and UCNP-GA control, respectively. Adapted with permission from Ref. [122], © Wiley 2019.

Polyphenol and metal ions complexes are also the best choice to fabricate pH-triggered films on the surface of multiple kinds of nanostructures to improve their biocompatibility and endow them some novel features [168]. For instance, Gao *et al*. [124] coated the upconversion nanoparticles with TA and Fe^3+^ to prepare the multifunctional tumor theranostic agents (UCNP@GA-Fe^III^) (Fig. 13A). The pH-responsive combination and decomposition of diphenol groups of TA and iron ions (Fig. 13B) under different conditions endows UCNP@GA-Fe^III^ amplified upconversion luminescence and magnetic resonance imaging signal in the acid tumor microenvironment (Fig. 13A and E). Surprisingly, the existence of iron ions on the surface of nanoparticles made them easily adsorb transferrin in the blood to enhance their tumor accumulation (Fig. 13C and E). Eventually, the synergistic effect of ferroptosis and thermal ablation in this study displayed a significant tumor growth inhibition behavior *in vivo* (Fig. 13D).

These studies provide the paradigm for fabricating the metal–polyphenol complexes to drive ferroptosis and improve traditional therapy manners of the tumor, such as chemotherapy, immunotherapy and photothermal therapy. We believe that more metal–polyphenol-based designable nanosystems will be developed in the future to regulate ferroptosis and improve cancer therapy.

##### Other metal-based complexes

Besides metal–polyphenol complexes, various metal-based nanostructures exhibited decomposition behaviors under acid conditions, such as iron oxide nanoparticles, calcium carbonate nanoparticles, MOFs and so on. The released metal ions from those metal-based complexes displayed outstanding catalytic performance to transform H_2_O_2_ to high toxic •OH in the tumor cells, such as Fe^2+^, Cu^2+^, Mn^2+^and so on. The produced •OH further aggravated the lipid peroxide to drive ferroptosis.

**Figure 14.**
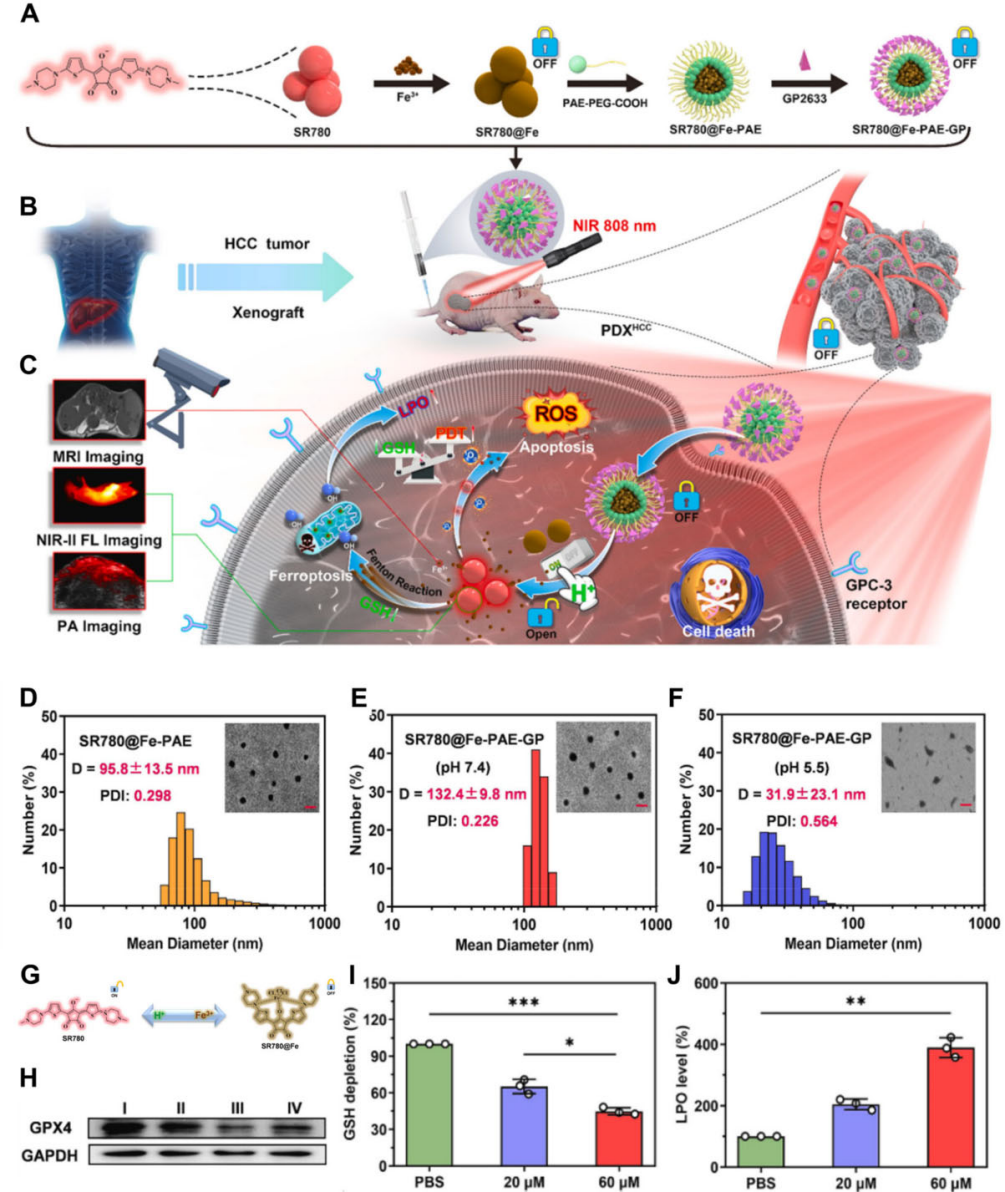
(**A**) Schematic representation of the preparation of pH-responsive active targeting nanoparticles with ‘off/on’ function. (**B** and **C**) The mechanism of photodynamic therapy combined with ferroptosis for cancer theranostics. (**D**–**F**) The size distribution of (D) SR780@Fe-PAE, (E) SR780@Fe-PAE-GP and (F) SR780@Fe-PAE-GP at pH 5.5 by DLS. Inset: the TEM photographs of SR780@Fe-PAE, SR780@Fe-PAE-GP and SR780@Fe-PAE-GP at pH 5.5 (Scale bar = 200 nm). (**G**) The structure conversion of SR780 and SR780@Fe in Fe^3+^ and acidic environments, respectively. (**H**) Western blot analysis of the expression of GPX4 protein in HepG2 cells treated with different formulations (I: PBS, II: SR780-PAE-GP, III: SR780@Fe-PAE, IV: SR780@Fe-PAE-GP). (**I**) Intracellular GSH in HepG2 cells treated with SR780@Fe-PAE-GP at different concentrations. (**J**) Intracellular LPO in HepG2 cells treated with SR780@Fe-PAE-GP at different concentrations. In (**I**) and (**J**), *P < 0.05, **P < 0.01, and ***P < 0.001. Adapted with permission from Ref. [126], © Elsevier 2022.

For example, Sun *et al*. [126] prepared an ‘off/on’ switchable nanoplatform (SR780@Fe-PAE-GP) to overcome the drug resistance of hepatocellular carcinoma and improve the multi-mode imaging signal (Fig. 14A–C). The amphiphilic SR780 dye was deactivated by Fe^3+^ and activated after the release of Fe^3+^ during the disintegration of the nanoplatforms in the mild acid environment (Fig. 14A and D–G). Ferroptosis was boosted by the released iron ion via significantly inhibiting the expression of GPX4 (Fig. 14H), and augmenting the depletion of GSH (Fig. 14I) and the production of lipid peroxide (Fig. 14J), which enhanced the PDT effect of activated SR780 dye. This synergistic therapy strategy achieved a 98% tumor growth inhibition rate *in vivo*.

Many other metal-based nanosystems regulate ferroptosis following similar mechanisms, and we didn’t list them one by one here. Such as Tang *et al*. [169] provide a kind of hollow mesoporous Prussian blue nanoparticles with pH-sensitive properties to deliver Sorafenib (SO), a ferroptosis accelerant agent, Ma *et al*. [127] fabricated a ‘core-shell’ magnetic nanosystems based on iron oxide and manganese oxide structures to boost ferroptosis via the Fenton reaction in cancer, and so on. Taking advantage of the pH stimuli-responsive metal-based nanoparticles, the function of metal ions and other components will be integrated into one smart nanoplatform. The multi-functional property of these kinds of nanoplatform, such as pH-triggered depletion of GSH, accumulation of ROS and drug release ability, will make them the promising tool to drive ferroptosis in the future.

#### Glucose-responsive nanosystems for inducing ferroptosis

With rapid metabolism and proliferation, tumor cells absorb considerable amounts of glucose, which is catalyzed by endogenous enzymes to generate gluconic acid and H_2_O_2_ [170, 171]. The catalytically generated H_2_O_2_ not only upregulates the level of ROS in tumor cells but also provides enough substrate for the Fenton reaction to amplify the generation of highly toxic •OH, which further promotes the effects of ferroptosis. In addition to endogenous enzymes, some nanozymes have the ability to respond to glucose and catalyze the production of H_2_O_2_ [172, 173]. Numerous studies have confirmed that ferroptosis in tumor tissues is induced or enhanced by ROS generated from glucose.

**Figure 15.**
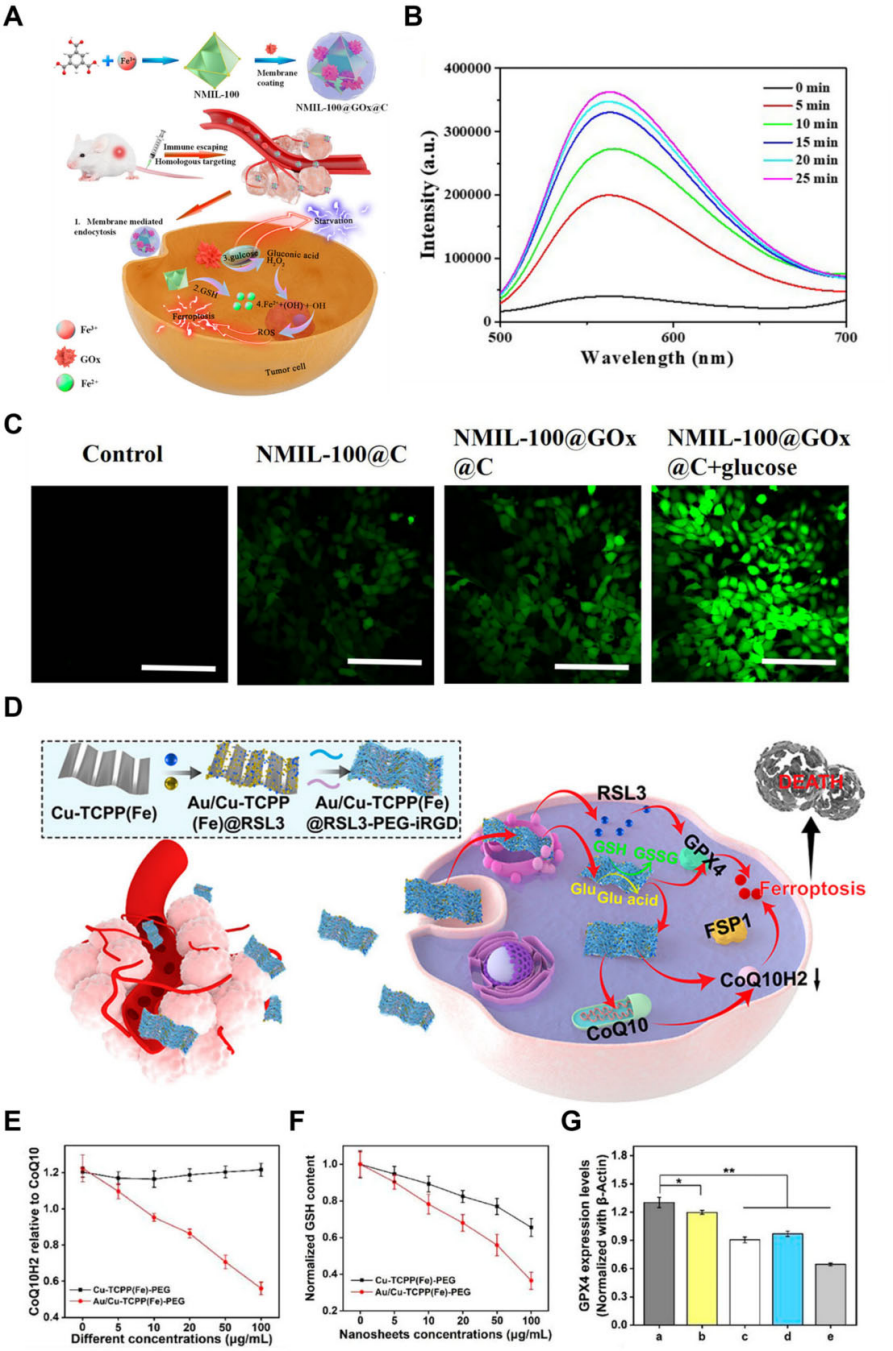
(**A**) Schematic illustration of the preparation of NMIL-100@GOx@C and the Cascade processes for cancer therapy. (**B**) Fluorescence intensity of ER-H_2_O_2_ in the NMIL-100@GOx@C solution at different time after addition of glucose. (**C**) CLSM images of 4T1 cells treated under different conditions to evaluate ROS production based on DCF-DA fluorescence intensity. Adapted with permission from Ref. [128], © American Chemical Society 2020. (**D**) Schematic illustration of the tumor-targeting composite nanosheet system for sensitizing tumor ferroptosis via impairing GPX4/GSH and FSP1/CoQ10H2 pathways. (**E**) Ratio of CoQ10H2 to CoQ10 in 4T1 cells. (**F**) Relevant intracellular GSH after Cu-TCPP(Fe)-PEG and Au/Cu-TCPP(Fe)-PEG treatment. (**G**) The GPX4 protein expression after different treatments, *P < 0.05, **P < 0.01, and ***P < 0.001. (**a**) Control; (**b**) Cu-TCPP(Fe)-PEG (10 μg/ml); (**c**) Cu-TCPP(Fe)-PEG (50 μg/ml); (**d**) Au/Cu-TCPP(Fe)-PEG (10 μg/ml); and (**e**) Au/Cu-TCPP(Fe)-PEG (50 μg/ml), *n* = 3. (D–G) Adapted with permission from Ref. [129], © American Chemical Society 2022.

Tang *et al*. [128] designed a nanoreactor based on glucose oxidase (GOx) to induce synergistic ferroptosis–starvation anti-cancer therapy. Briefly, the authors synthesized a GOx-loaded iron-based MOF (NMIL-100) coated with a cancer cell membrane (Fig. 15A). NMIL-100, played the role of iron source of ferroptosis and carrier, disintegrated in high concentration-GSH environment to release Fe^2+^ and GOx due to the reduction effect of GSH on Fe^3+^. Subsequently, the released GOx catalyzed glucose to generate amounts of H_2_O_2_ (Fig. 15B), which produced a large amount of •OH through the Fenton reaction under the action of Fe^2+^ to enhance the overall ROS level for ferroptosis therapy (Fig. 15C).

Based on the properties of mimic enzymes, Au NPs can be used as GOx to oxidize glucose [129]. For example, Li *et al*. reported an enzyme-like nanosheet, Au/Cu-TCPP(Fe)@RSL3-PEG-iRGD (Fig. 15D). In this nanosheet, the Au NPs exhibited GOx-like activities to catalyze the oxidation of glucose to gluconic acid and H_2_O_2_ to upregulate ROS levels and efficient glucose depletion, which impeded GSH biosynthesis by disturbing the pentose phosphate pathway and preventing the conversion of coenzyme Q10 (CoQ10) into CoQ10H2 (Fig. 15E). GSH was rapidly oxidized by Cu^2+^ to form GSSG (oxidized glutathione) (Fig. 15F). In addition, the released RSL3 reduced the activity of the GPX4 protein, thus reducing the activity of GPX4 from multiple perspectives (Fig. 15G). In conclusion, this nanosheet system developed a glucose-responsive strategy to achieve tumor ferroptosis sensitization by impairing the GPX4/GSH and FSP1/CoQ10H2 pathways.

The H_2_O_2_ produced during glucose catalysis is an important source of ROS for ferroptosis. More importantly, depletion of glucose hinders the synthesis of GSH, leading to a decrease in the activity of GPX4. Furthermore, it prevents CoQ10 from producing CoQ10H2, which is involved in the capture of lipid peroxide free radicals in the FSP1-mediated antiferroptosis pathway for detoxification of lipid peroxides. Consequently, glucose-responsive nanomaterials can enhance ferroptosis from multiple perspectives and contribute to the development of more advanced ferroptosis-based anti-tumor drugs.

## Conclusions and future prospective

The application of ferroptosis in cancer therapy has received great attention and has been widely reported since it was first proposed in 2012. In recent years, ferroptosis has always been considered a fascinating research subject, prompting many researchers to study it, aiming to develop effective anti-cancer strategies based on it. Studies based on ferroptosis inducers have expressed their promising anti-cancer potential. Rapid nanotechnological advancements in the medical field have broadened the class of ferroptosis inducers and facilitated their development. Moreover, owing to their unique physicochemical properties, responsive nanomaterials have many advantages in improving the therapeutic effect of small-molecule inducers and giving better opportunities for inducing ferroptosis for cancer therapy. Taking advantage of these, a series of stimuli-responsive nanomaterials have been designed to induce ferroptosis for cancer therapy. Therefore, in this review, we introduced stimuli-responsive nanomaterials that induced ferroptosis for cancer therapy, particularly focusing on external stimuli and tumor internal stimulus responses. While numerous studies have been conducted on ferroptosis inducers and their applications based on various stimuli-responsive nanomaterials, there are still some limitations, difficulties, unresolved questions and areas that require more in-depth research and discussion to improve the existing materials or design a new one. The following are some perspectives on the development and challenges of this research.

First, different external stimulation therapies present different difficulties and challenges. For instance, the side effects of ionizing radiation are the main problems that need to be solved in conventional RT. Owing to their high X-ray absorption, the use of high-Z metal nanomaterials as X-ray sensitizers to effectively reduce the radiation dose is a feasible approach [174, 175]. In the case of light-triggered PDT, inefficient penetration of external stimuli is the main challenge to therapy efficacy, limiting the application of ferroptosis as a ROS source for deep tumors. Near-infrared (NIR) light penetrates deeper (∼1 cm) than ultraviolet (UV) light (∼10 mm); therefore, designing nanomaterials that are activated in this wavelength range is a method to overcome the limited penetration depth. In addition, two-photon technology, which converts UV light sources into light sources in the NIR region, is also a promising method for achieving a better therapeutic effect of PDT [176]. Ultrasound-mediated SDT, with similar flaws as PDT, may be ameliorated by organelle-targeted sonosensitizers. Furthermore, overcoming the effects of photosensitizers or sonosensitizers on the normal tissue around the tumor is another issue that researchers must consider.

Moreover, the hypoxic tumor microenvironment limits the anti-tumor effects of PDT and SDT, based on external stimuli. Both PDT and SDT are O_2_-dependent therapies, because ROS are generated by the interaction of molecular oxygen with photoactivated photosensitizers and ultrasound-activated sonosensitizers, respectively. Consequently, the therapeutic effects of PDT or SDT ferroptosis are limited by the oxygen concentration inside the tumor. Currently, the existing methods for overcoming hypoxia are roughly divided into oxygen-carrying and oxygen-generating strategies [177, 178]. More importantly, these hypoxia-improving strategies could be applied to modulate ferroptosis based on PDT or SDT in the future.

Then, various responsive chemical bonds are key to internal stimuli-responsive nanosystems. The response sensitivity of the chemical bonds guarantees the precise drug release of each nanosystem. Therefore, improving response sensitivity and choosing the appropriate type of chemical bonds is worthy of consideration and research. In addition, the factor that affects the effectiveness of stimuli-responsive nanoplatforms is the accurate synthesis of nanomaterials. As ferroptosis-inducing agents based on internal stimuli-responsive nanodelivery systems have only recently attracted attention, there are relatively few studies and reports in this regard. In the future, it is expected that the types of chemical bonds used to regulate ferroptosis will become more abundant.

Finally, the interaction of NPs with biological systems is complex with various physical and chemical processes, influencing the fate of NPs and determining the final effect of ferroptosis [179, 180]. Therefore, diverse nano-bio interface interactions should be seriously considered and discussed. One of the important issues is how NPs interact with environmental molecules, such as proteins [181–183]. Wang *et al*. [184, 185] developed a series of synchrotron radiation analytical techniques to elucidate the valence of metal elements in various conditions and illustrate the interaction between NPs and proteins [186, 187]. Besides, they comprehensively provided quantitative methods to monitor the content and transformation of NPs in the blood and different tissues [188, 189]. The application of a methodology for the interaction and fates in nano-bio science is crucial to understand the safety and anti-tumor effect of NPs, helping explore the mechanism of ferroptosis. Therefore, it will be a promising research orientation in the near future.

As an emerging and promising therapeutic method, ferroptosis has attracted increasing attention for cancer treatment. Moreover, ferroptosis provides new targets and design ideas for the diagnosis and treatment of tumors, which is an important research topic in the future. Responsive nanomaterials offer many advantages in inducing ferroptosis, and it is hoped that this review will provide guidance on the design of stimuli-responsive nanomaterials to modulate ferroptosis.
